# How Does Gender Stereotype Affect the Memory of Advertisements? A Behavioral and Electroencephalography Study

**DOI:** 10.3389/fpsyg.2020.01580

**Published:** 2020-07-14

**Authors:** Shih-Yu Lo, Jung-Tai King, Chin-Teng Lin

**Affiliations:** ^1^Institute of Communication Studies, National Chiao Tung University, Hsinchu, Taiwan; ^2^Center for General Education, National Chiao Tung University, Hsinchu, Taiwan; ^3^Institute of Neuroscience, National Yang-Ming University, Taipei, Taiwan; ^4^CIBCI Lab, Australia Artificial Intelligence Institute (AAII), FEIT, University of Technology Sydney, Sydney, NSW, Australia

**Keywords:** gender stereotype, advertising effect, memory recognition, event-related potentials, parietal old/new effect

## Abstract

Previous studies have shown equivocal results about whether atypical or unusual events, compared with typical ones, facilitate or inhibit memory. We suspect that the indefinite findings could be partly due to the recall task used in these studies, as the participants might have used inference instead of recall in their responses. In the present study, we tested the recognition memory for real (Experiment 1) and fabricated (Experiment 2) advertisements, which could be congruent or incongruent with gender stereotypes. In congruent advertisements, a female endorser presented a traditionally considered feminine product or a male endorser presented a traditionally considered masculine product, whereas the gender-product type matching reversed in incongruent advertisements. The results of both behavioral experiments revealed that the participants’ memory performance for stereotype-incongruent advertisements was higher than for congruent ones. In the event-related potential (ERP) recordings in Experiment 3, larger positive amplitudes were found for stereotype-incongruent advertisements than for congruent advertisements on the left parietal sites, suggesting a deeper encoding process for stereotype-incongruent information than for stereotype-congruent information.

## Introduction

The cognitive system creates mental representations of external objects or events that allow us to make sense of the world. Each representation can be viewed as a *concept*. These concepts can be understood as the building blocks of our knowledge systems, and the way in which these miscellaneous concepts are organized or sorted is often referred to as a *schema*. The introduction of schema has inspired a series of behavioral and neuro-scientific studies (Alba and Hasher, 1983; van Kesteren et al., 2012) as well as the theoretical development of artificial intelligence and connectionist modeling (McClelland et al., 1995).

### Schema-Congruency Effect on Memory

In the cognitive system, memories inconsistent with one’s schema can be successfully retrieved more frequently, as demonstrated in a recognition task (Graesser et al., 1980; Schmidt, 1991; Erdfelder and Bredenkamp, 1998). In layperson’s terms, unusual or atypical events are inherently more memorable than usual or typical events. One explanation is that mental schemas lead to shallow coding of goal-irrelevant information (Sweegers and Talamini, 2014; Sweegers et al., 2015). When stimuli are not firmly encoded owing to insufficient presentation time (Vilberg and Rugg, 2009), attention (Craik et al., 1996; Naveh-Benjamin et al., 2003), or repetition (Nelson, 1977; Rugg and Doyle, 1994), the memory traces decay. Schema-incongruent information requires more effort to encode, resulting in deeper processing and thus better retrieval.

When schemas are applied to social contexts, they often lead to *stereotypes*. For example, the concept of “doctor” is often related to other concepts such as “white robe,” “middle-aged,” and “male.” All of these interconnected concepts constitute both the schema and the stereotype for “doctor.” Schema-incongruent information not only requires more effort to encode but also requires more cognitive resources, resulting in a memory benefit for this information. For example, in one study conducted by Kroneisen and Bell (2013), participants were required to remember a series of human faces associated with personality descriptions. They found that female faces could be recognized more frequently if they were paired with cheating descriptions. The rationale was that men, compared with women, were more likely to be associated with the cheating quality, and thus women associated with cheating descriptions were more memorable.

### Counter-Examples of the Schema-Incongruent Memory Benefit

Some studies showed a memory benefit for stereotype-congruent information instead of stereotype-incongruent information. In one study, participants viewed a series of 12 consecutively presented pictures including ethnic Africans and Europeans. Participants were told that these people attended auditions for roles as teachers, artists, or drug dealers. Twenty minutes later, they had to recall the roles for which these people auditioned. The results showed that the participants tended to recall the roles that were consistent with their racial stereotypes (Kleider et al., 2012). Similarly, participants in Castel’s (2005) study were instructed to remember a list of grocery items and their prices. The results showed that participants could recall the prices more often if the prices were closer to their expectations. It is possible that in a recall task, participants are more likely to infer their answers, and thus the schema-congruent memory benefit could in fact be a schema-incongruent *inference* benefit. In the study conducted by Kleider et al. (2012), participants inferred the role for which an individual was supposed to audition, resulting in more reports of roles that were congruent with the participants’ stereotypes; in the study performed by Castel (2005), participants inferred the price of a grocery item, resulting in more reports of prices that were congruent with the participants’ expectations.

### Applications of Schemas

Basic research on memory is important, not only for fulfilling our curiosity about human cognition but also for identifying potential applications of the persuasion techniques. A major aim of advertising is to enhance consumers’ positive attitude toward a particular brand. A very commonly used strategy is to associate the brand with a celebrity endorser (Choi and Rifon, 2012; del Mar Garcia de los Salmones et al., 2013). One stream of studies showed that a match between an endorser and brand led to better communication effects (Kamins, 1990; Till and Busler, 2000; Lee and Thorson, 2008), as a congruent context helps potential consumers understand the advertising (Goodstein, 1993; Kamins and Kamal, 1994). Another line of research showed an opposite effect: Mismatch between the endorser and brand improved purchase intensions (Debevec and Iyer, 1986; Ryu et al., 2006; Törn, 2012; Harmon-Kizer, 2017), perhaps by inducing curiosity and interest in the brand and in turn enhancing the consumer’s positive attitude. The equivocal effects of schema congruency on a consumer’s attitude could be due to the multifaceted nature of attitude. According to the ABC model (Eagly and Chaiken, 1998; Van den Berg et al., 2006), attitude is composed of three components: affect, behavior, and cognition. The different measurements of attitude in the aforementioned studies might reflect different components of attitude.

Memory of a brand could be a predictor of one’s attitude for the brand (Malodia et al., 2017), and therefore, an important aim of advertising is to impress consumers. Psychological studies have demonstrated that stereotype incongruency has a benefit for memory in general (Graesser et al., 1980; Schmidt, 1991; Erdfelder and Bredenkamp, 1998), and this effect should be generalizable to memory of advertisements. However, in reality, stereotype-congruent advertisements are more prevalent than incongruent ones. For example, regarding gender stereotypes, a content analysis of Taiwanese commercials indicated that stereotype-congruent messages were more prevalent than stereotype-incongruent ones (Tsai, 2010). As advertisements could influence gender identity-related concepts (Schroeder and Borgerson, 1998), a potential consequence of propagating stereotypical images in the media is reinforcement of the stereotype, which might result in stereotype threat (Deaux et al., 2007; Schmader et al., 2008).

### Aim of the Present Study

In this study, we aimed to empirically test whether gender-stereotypical messages in advertisements could enhance or suppress memory and purchase intention. We used the term “gender” instead of “sex” because “stereotype” is a socio-psychological construct and thus is more applicable to studies on stereotypes. Given that the effects of schema congruency on memory probed in previous studies could be confounded with a schema-congruent *inference* benefit instead of a schema-congruent *memory* benefit, a recognition task was used in the present study. In the training stage, participants viewed a series of advertisements, and in the testing stage, participants had to identify whether or not a particular advertisement was an old item (i.e., they had seen it in the training stage) or a new item (i.e., they had not seen it in the training stage).

## Experiment 1

This experiment was designed to test how gender stereotypes affect memory. A series of advertisement pictures with products and endorsers were presented to the research participants. Traditionally considered feminine products endorsed by females or traditionally considered masculine products endorsed by males were considered to be “stereotype congruent,” and other cases were considered to be “stereotype incongruent.”

Previous studies on attention have shown that attention plays an important role in selecting information for conscious reports (Goodbourn and Holcombe, 2015; Lo and Wang, 2019). Therefore, it was important to consider attention in our experimental design. A substantial amount of studies have postulated a two-stage model of visual attention (Kanwisher, 1987; Chun and Potter, 1995; Bowman and Wyble, 2007; Goodbourn and Holcombe, 2015; Lo and Yeh, 2018; Lo and Wang, 2019). In the first stage, the visual system can only create a *type* representation of the visual object, which is fleeting and inaccessible for conscious report. A second stage of processing is required to consolidate the type representation into a *token* representation, based on which the observer to make a conscious report. The second stage takes place approximately 200–500 ms after stimulus onset (Raymond et al., 1992; Chun and Potter, 1995). We used two presentation times, 83 and 976 ms, which align with the first and second stages of visual attention processing, respectively.

### Method

#### Participants

The experiments in this study were approved by the Research Ethics Committee for Human Subject Protection at National Chiao Tung University (Case number: NCTU-REC-106-027), and the participants in all the experiments gave their written consent before participating. All the participants had normal or correct-to-normal vision, and they reported no neurological or psychopathological disorders.

To determine the sample size, we used the study of Kroneisen and Bell (2013) as a reference. In this study, the researchers used the *Pr* value (a sensitivity index for recognition performance) to analyze how gender stereotype affected memory of faces, and they found an interaction between gender and personality description for a face with an effect size of η^2^_partial_ = 0.08. In other words, the congruency between gender and personality descriptions accounted for 8% of the variance of *Pr* for a face. We thus used an η^2^_partial_ of 0.08 and set α as 0.05 in order to compute the required sample size with the software program G^∗^Power 3 (Faul et al., 2007)^1^. To achieve a power of 0.8, a minimum sample size of 25 was required.

As presentation time was manipulated as a between-subject factor with two levels (83 and 977 ms), we aimed to recruit at least 50 people so each group could contain at least 25 people. Before we closed the recruitment advertisements, 62 people (31 females) aged approximately 20–25 years (*M* = 22, *SD* = 2) had signed up, and we included them all.

#### Procedure

The experiment was programmed in MATLAB r2014b (32-bit) with Psychtoolbox-3 extensions (Brainard, 1997; Pelli, 1997). Visual stimuli were displayed on a 17-in. CRT monitor (Mitsubishi i-TECH IF700 CRT Monitor) with a spatial resolution of 1,024 × 768 pixels and a refresh rate of 85 Hz. Each participant was seated in a dimly lit chamber with a viewing distance of approximately 66 cm.

The participants were explicitly told that they had to complete a memory task. After providing their demographic data including the self-identified gender and age, they started the practice session. In the practice training stage, the participants viewed two pictures. In the practice testing stage, they viewed four pictures and had to judge whether they were presented in the practice training stage. The presentation times for the stimuli in the practice session were identical to those in the experimental session, but all the pictures were different, and those used in the practice session did not contain any information about gender stereotypes.

All the advertisements used in the experimental session were drawn from a pool of 80 advertisements: 4 for perfumes, 12 for scooters, 16 for shampoos, 16 for alcohol, 4 for cars, 8 for facial creams, 4 for rice cookers, 8 for laundry powders, and 8 for phones. For each product type, half were endorsed by females and the other half were endorsed by males. Thus, there were a total of 40 stereotype-congruent advertisements and 40 stereotype-incongruent advertisements. All the advertisements used in this experiment were downloaded in October 2017 after using the product names (e.g., perfume) as keywords in a Google search.

**FIGURE 1:**
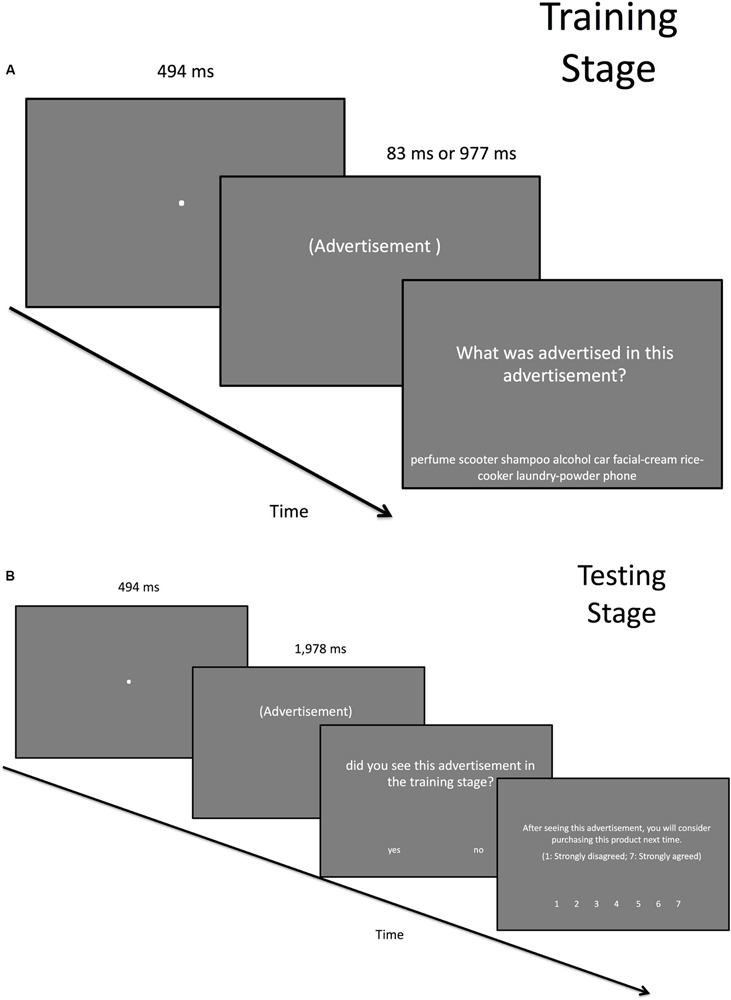
Stimuliused in the **(A)** training stage and **(B)** testing stage. Note that the actual advertisement is not shown here owing to potential copyright issues. All the instructions were shown in Chinese in the actual experiment.

The experimental session comprised the training and testing stages. In the training stage, the participants viewed 40 advertisements drawn from the aforementioned pool, of which 20 were stereotype congruent and 20 were stereotype incongruent. The trial began with a fixation display that contained a white disk with a diameter of 10 pixels on a gray background and lasted for 494 ms. Then, an advertisement was presented for either 83 (30 participants) or 977 ms (32 participants). Participants were randomly assigned into either the 83-ms condition or the 977-ms condition. After the advertisement display came the product inquiry display, in which a question “What was advertised in this advertisement?” was shown in the upper field of the display. In the lower field of the display, there were nine product options: perfume, scooter, shampoo, alcohol, car, facial cream, rice cooker, laundry powder, and phone. Participants had to use the mouse to click on the product type (Figure 1A).

The ending of the training stage was followed by a 20-s break. In the study conducted by Kroneisen and Bell (2013), a 20-s break between the training stage and the testing stage was sufficient for the gender stereotype to exert an effect on face memory, so we followed their procedure. After the break came the testing stage, which was composed of 80 trials. Each testing trial began with a 494-ms fixation display, as in the training stage, followed by a 1,978-ms advertisement display. In half of the trials, the advertisement display contained an advertisement that the participant had viewed in the training stage, and in the other half, the advertisement display contained a new advertisement. Following the advertisement display, the recognition inquiry display was presented. A question was shown in the upper field “Did you see this advertisement in the training stage?” and the participant had to click on either “yes” and “no,” which were shown in the lower field of the display. Next was the purchase intention inquiry display. In the upper field, a question was shown “After seeing this advertisement, you will consider purchasing this product next time (1: strongly disagree; 7: strongly agree)”; and in the lower field, the numbers 1–7 were shown. The participant had to click on a number to indicate their intention to purchase the product (Figure 1B).

After each trial in the training and testing stages was completed, a progress feedback display showed how many trials had been completed. This provided the participants with knowledge of their own progress, making it possible for them to adjust their pace so they could finish the experiment within 1 h. After viewing the progress feedback, the participants clicked the mouse to initiate the next trial. All the verbal instructions described above were printed in white on a gray background.

#### Manipulation Check

A questionnaire was designed to test the strength of the association between the product type and the endorser’s gender. In this questionnaire, the nine product types were listed, and a group of respondents who did not participate in the experiment indicated how much each product type was associated with women or men on a 7-point Likert scale in which −3 indicated very masculine, 3 indicated very feminine, and 0 indicated neutrality. The rating results across the 94 respondents indicated that the scooter (*t*[93] = 8.92, *p* < 0.001), alcohol (*t*[93] = 11.82, *p* < 0.001), and car (*t*[93] = 16.88, *p* < 0.001) were perceived as more related to men, whereas perfume (*t*[93] = 15.16, *p* < 0.001), shampoo (*t*[93] = 9.47, *p* < 0.001), facial cream (*t*[93] = 19.16, *p* < 0.001), rice cooker (*t*[93] = 10.18, *p* < 0.001), and laundry powder (*t*[93] = 11.78, *p* < 0.001) were perceived as more related to women. The phone (*t*[93] = 0, *p* > 0.99) was perceived to be gender neutral, as indicated by the mean rating of exactly 0. In the main task, participants still viewed phone advertisements, but all the trials with phone advertisements were removed for statistical analysis, as described below.

### Data Analysis

We used two criteria to determine which data would be subjected to further analysis. First, the accuracy had to exceed a chance level of 50%. Second, based on our calculations of the interquartile ranges for the 83-ms group and the 977-ms group, the accuracy for each participant had to not be lower than the lower outlier boundary for their group (which was lower than the first quartile by 1.5 times the interquartile range). On the basis of the two criteria, one participant in the 977-ms condition was removed, as the accuracy value was lower than the lower outlier boundary. Of the remaining participants, 31 (16 females) participated in the 977-ms condition and 30 (14 females) participated in the 83-ms condition.

Memory performance was indexed by *Pr* (Bell et al., 2010; Kroneisen and Bell, 2013) based on the two-high threshold model. The usage of *Pr* as a sensitivity measure was validated by Snodgrass and Corwin (1988). In this study, the *Pr* value was calculated by subtracting the false alarm rate from the hit rate. A hit response was defined as occurring when the participant correctly provided a “yes” response to an advertisement that had been presented in the training stage, and a false alarm response was defined as occurring when the participant incorrectly provided a “yes” response to an advertisement that had not been presented in the training stage.

### Results

#### Recognition Performance

**FIGURE 2:**
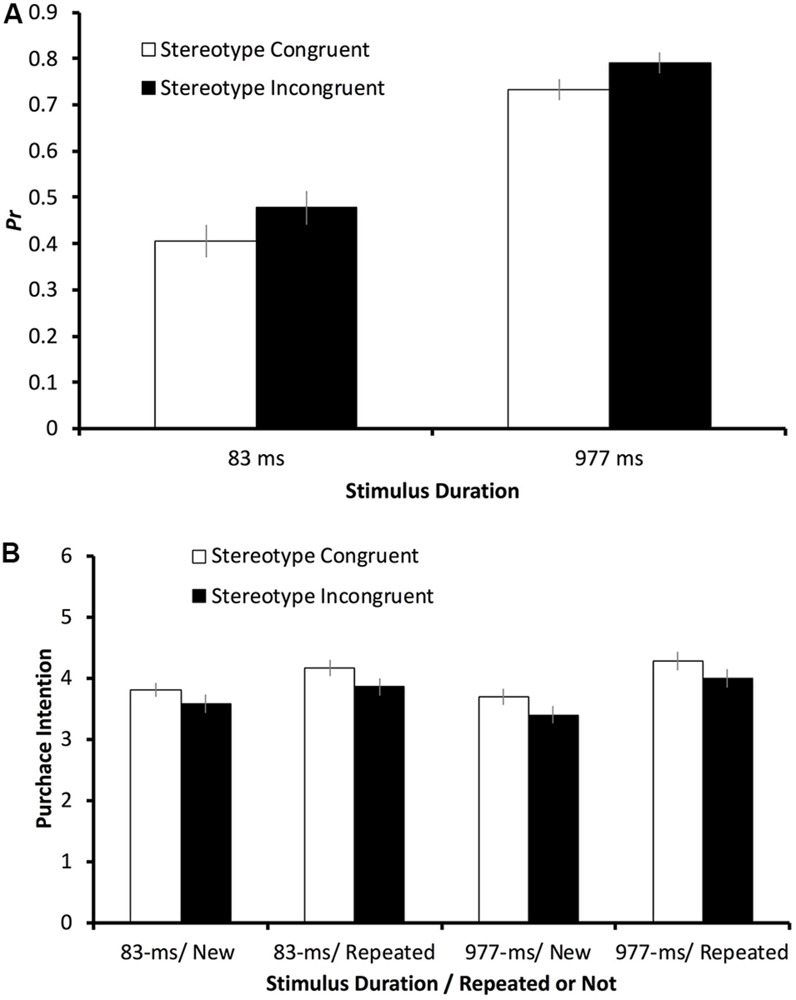
The **(A)** *Pr* values and **(B)** purchase intention ratings in different conditions of Experiment 1. The error bars indicate one standard error.

In general, the participants’ ability to recognize the advertisements was higher for the incongruent advertisements than for the congruent advertisements. The mean discriminability index *Pr* values (Figure 2A) were 0.73 and 0.79 for the congruent and incongruent advertisements, respectively, in the 977-ms condition, and 0.40 and 0.48 for the congruent and incongruent advertisements, respectively, in the 83-ms condition. The data were subjected to a mixed-design analysis of variance (ANOVA) with one within-subject factor (Congruency: stereotype congruent vs stereotype incongruent) and two between-subject factors (Duration and Participant Gender).

The effect of Duration reached significance (*F*[1, 57] = 88.30, *p* < 0.001, η^2^_partial_ = 0.61) owing to the significantly higher *Pr* value in the 977-ms condition than the 83-ms condition. The effect of congruency was significant as well (*F*[1, 57] = 8.30, *p* = 0.006, η^2^_partial_ = 0.13) caused by the significantly higher *Pr* value in the incongruent condition compared with the congruent condition. Participant Gender did not yield a significant effect (*F*[1, 57] = 3.14, *p* = 0.08, η^2^_partial_ = 0.05), and it did not significantly interact with Duration (*F*[1, 57] = 1.39, *p* = 0.24, η^2^_partial_ = 0.02) or Congruency (*F*[1, 57] = 0.59, *p* = 0.45, η^2^_partial_ = 0.01). There was no significant interaction between Duration and Congruency (*F*[1, 57] = 0.12, *p* = 0.73, η^2^_partial_ = 0.002) or among the three factors (*F*[1, 57] = 0.45, *p* = 0.51, η^2^_partial_ = 0.008).

#### Purchase Intention

All of the advertisements the participants had viewed in the testing stage were included in the purchase intention analysis. Half of the advertisements were viewed twice because they had been presented in the training stage, and the other half were viewed only once because they were new to the participants in the testing stage. The participants’ mean purchase intention ratings, measured by a 7-point Likert scale, are shown in Figure 2B.

The data were then subjected to a mixed-design ANOVA in which Repetition was included as an additional factor. A significant effect was found for Congruence (*F*[1, 57] = 36.02, *p* < 0.001, η^2^_partial_ = 0.39), meaning that participants’ intention to purchase a product from a stereotype-congruent advertisement was higher than for a stereotype-incongruent one. In general, the participants’ intention to purchase a product was higher for a repeated advertisement than a new one, as the main effect of Repetition was significant (*F*[1, 57] = 47.29, *p* < 0.001, η^2^_partial_ = 0.45). Additionally, this repetition benefit was higher for the 977-ms condition than the 83-ms condition, as the interaction between Duration and Repetition also reached statistical significance (*F*[1, 57] = 4.13, *p* = 0.047, η^2^_partial_ = 0.07).

There were several non-significant effects. Participant Gender did not yield a significant effect (*F*[1, 57] = 3.17, *p* = 0.08, η^2^_partial_ = 0.05), and it did not interact with Duration (*F*[1, 57] = 0.58, *p* = 0.45, η^2^_partial_ = 0.01), Congruency (*F*[1, 57] = 2.22, *p* = 0.14, η^2^_partial_ = 0.04), or Repetition (*F*[1, 57] = 0.34, *p* = 0.56, η^2^_partial_ = 0.006). The interactions between Congruency and Repetition (*F*[1, 57] = 0.30, *p* = 0.59, η^2^_partial_ = 0.005) and between Congruency and Duration (*F*[1, 57] = 0.04, *p* = 0.85, η^2^_partial_ = 0.0006) did not reach significance. In addition, significance was not reached for any of the three-way interactions (Duration, Congruency, Participant Gender: *F*[1, 57] = 0.78, *p* = 0.38, η^2^_partial_ = 0.01; Duration, Congruency, Repetition: *F*[1, 57] = 0.36, *p* = 0.55, η^2^_partial_ = 0.006; Congruency, Participant Gender, Repetition: *F*[1, 57] = 0.009, *p* = 0.92, η^2^_partial_ = 0.0001; Duration, Participant Gender, Repetition: *F*[1, 57] = 0.29, *p* = 0.59, η^2^_partial_ = 0.005), or the four-way interaction (*F*[1, 57] = 0.31, *p* = 0.58, η^2^_partial_ = 0.005).

### Discussion

The old/new discriminability index *Pr* value was higher for stereotype-incongruent advertisements than stereotype-congruent ones. This could be explained by the fact that the stereotype-incongruent advertisements were less typical, so they might be more “unforgettable” for the participants.

Interestingly, stereotype congruency had the opposite effects on memory performance and purchase intention: For memory, there was a stereotype-incongruence benefit, and for purchase intention, there was a stereotype-congruence benefit. The finding that higher purchase intention was associated with stereotype-congruent products is consistent with previous studies, which demonstrated a better communication effect in the presence of matches between the endorser’s gender and the brand (Kamins, 1990; Till and Busler, 2000; Lee and Thorson, 2008). According to the theory of planned behavior (Ajzen, 1985) and the theory of reasoned action (Fishbein, 1976), the determinants for one to execute a certain behavior are attitude and subjective norms. The participants in the study might have been immersed in a media environment filled with gender stereotype images that shaped their attitudes and subjective norms. It is possible that, as a consequence, they demonstrated a preference for gender stereotype-congruent advertisements.

## Experiment 2

A potential issue in Experiment 1 was that the experimental materials were not very strictly controlled, as they were downloaded from the Internet. Therefore, the endorser’s popularity might have been a confounding factor. To address this potential issue, the same experiment was conducted again in Experiment 2, but all the advertisements were synthesized with pictures downloaded from online image database, and none of the endorsers were celebrities.

### Method

#### Participants

In this experiment, 60 people (29 females) aged approximately 20–25 years (*M* = 22; *SD* = 2) were recruited.

### Procedure

The procedure was identical to Experiment 1, except in a few ways. First, all the advertisements were created specifically for the experiment, and none of the endorsers were celebrities. All of the images were downloaded from the Shutterstock image database^2^ (see Supplementary Data Sheet S1). Second, as the phone was not associated with either males or females, no advertisements were created for the phone. Each type of advertisements (congruent and incongruent) was created: two for perfumes, six for scooters, eight for shampoos, eight for alcohols, six for cars, four for facial creams, two for rice cookers, and four for laundry powders.

### Data Analysis

We used the identical analysis method as that in Experiment 1.

### Results

#### Recognition

Two participants in the 83-ms condition were excluded from further analysis because their overall accuracy values were not significantly greater than a chance level of 50% (*z* = 1.45, *p* = 0.07). In addition, a participant in the 977-ms condition was removed because the accuracy value was lower than the lower outlier limit. Of the remaining participants, 28 (16 females) participated in the 83-ms condition and 29 (10 females) participated in the 977-ms condition.

**FIGURE 3:**
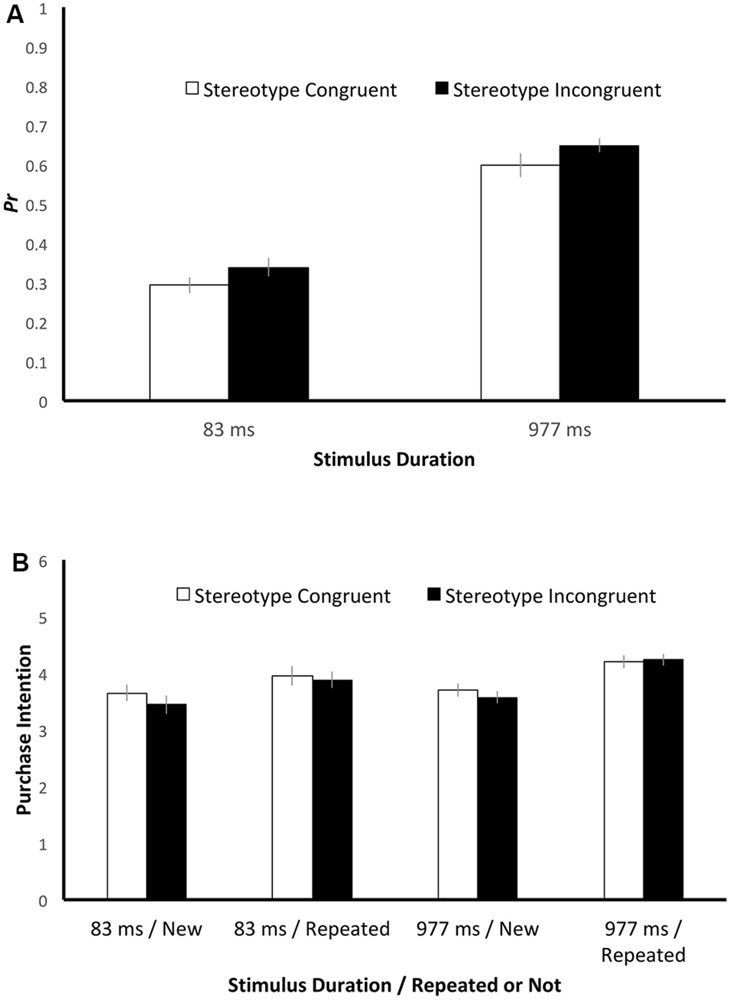
The **(A)** *Pr* values and **(B)** purchase intention ratings in different conditions of Experiment 2. The error bars indicate one standard error.

The mean *Pr* values (Figure 3A) were 0.29 and 0.34 for stereotype-congruent and stereotype-incongruent advertisements, respectively, in the 83-ms condition and 0.60 and 0.65 for the stereotype-congruent and stereotype-incongruent advertisements, respectively, in the 977-ms condition. The *Pr* values were subjected to a mixed-design ANOVA with one within-subject factor (Congruency: stereotype congruent vs stereotype incongruent) and two between-subject factors (Duration and Participant Gender). The effect of Duration reached significance (*F*[1, 53] = 154.18, *p* < 0.001, η^2^_partial_ = 0.74), as did the effect of Congruency (*F*[1, 53] = 4.50, *p* = 0.04, η^2^_partial_ = 0.08). For Duration, the participants’ ability to discriminate between an old item and a new one was higher in the 977-ms condition than in the 83-ms condition. For Congruency, the participants’ ability to discriminate between an old item and a new one was higher for stereotype-incongruent advertisements than for stereotype-congruent advertisements.

None of the other main effects or interactions reached statistical significance. Participant Gender did not yield a significant effect (*F*[1, 53] = 0.82, *p* = 0.37, η^2^_partial_ = 0.02). There was no significant interaction between Participant Gender and Duration (*F*[1, 53] = 3.83, *p* = 0.06, η^2^_partial_ = 0.07), between Participant Gender and Congruency (*F*[1, 53] = 0.17, *p* = 0.68, η^2^_partial_ = 0.003), or between Duration and Congruency (*F*[1, 53] = 0.006, *p* = 0.94, η^2^_partial_ < 0.001). There was no significant three-way interaction (*F*[1, 53] = 1.07, *p* = 0.30, η^2^_partial_ = 0.02).

#### Purchase Intention

Details about purchase intention are shown in Figure 3B. In addition to the factors of Congruency, Duration, and Participant Gender, the factor of Repetition was included in the ANOVA. There was a significant effect of Repetition (*F*[1, 53] = 69.40, *p* < 0.001, η^2^_partial_ = 0.57) caused by a higher purchase intention for repeated items compared with new items. The effect of Congruency was not significant (*F*[1, 53] = 2.86, *p* = 0.10, η^2^_partial_ = 0.05), with a trend of higher preference ratings for stereotype-congruent advertisements than stereotype-incongruent ones.

None of the other main effects or interactions reached statistical significance. There was no significant effect of Participant Gender (*F*[1, 53] = 0.39, *p* = 0.54, η^2^_partial_ = 0.007) or Duration (*F*[1, 53] = 1.31, *p* = 0.26, η^2^_partial_ = 0.02). There was no significant two-way interaction between Duration and Congruency (*F*[1, 53] = 0.79, *p* = 0.38, η^2^_partial_ = 0.01), Duration and Repetition (*F*[1, 53] = 3.37, *p* = 0.07, η^2^_partial_ = 0.06), Duration and Participant Gender (*F*[1, 53] = 0.97, *p* = 0.33, η^2^_partial_ = 0.02), Congruency and Repetition (*F*[1, 53] = 3.27, *p* = 0.08, η^2^_partial_ = 0.06), Congruency and Participant Gender (*F*[1, 53] = 0.78, *p* = 0.38, η^2^_partial_ = 0.01), or Repetition and Participant Gender (*F*[1, 53] = 0.19, *p* = 0.66, η^2^_partial_ = 0.004). There was no three-way interaction between Duration, Congruency, and Participant Gender (*F*[1, 53] = 0.16, *p* = 0.69, η^2^_partial_ = 0.003); Duration, Congruency, and Repetition (*F*[1, 53] = 0.04, *p* = 0.84, η^2^_partial_ < 0.001); Congruency, Participant Gender, and Repetition (*F*[1, 53] = 1.77, *p* = 0.19, η^2^_partial_ = 0.03); or Duration, Repetition, and Participant Gender (*F*[1, 53] = 1.53, *p* = 0.22, η^2^_partial_ = 0.03). In addition, there was no significant four-way interaction (*F*[1, 53] = 0.24, *p* = 0.63, η^2^_partial_ = 0.004).

### Discussion

In this experiment, all the experimental materials were lab-created specifically for the experiment. This was done so that the participants’ responses should not be confounded with their familiarity toward the celebrity endorsers or the product. The same pattern of results as Experiment 1 was observed again. The old/new discriminability index *Pr* was higher for stereotype-incongruent advertisements than stereotype-congruent advertisements. With regard to purchase intention, there was still a trend of higher purchase intention for the stereotype-congruent advertisements than for the stereotype-incongruent ones. However, the effect size did not reach statistical significance. A cross-experimental analysis of Experiments 1 and 2 showed a significant interaction between Experiment and Congruency (*F*[1, 116] = 7.53, *p* = 0.007, η^2^_partial_ = 0.06). The effect of gender stereotype on purchase intention was significantly reduced for unknown endorsers in comparison with celebrity endorsers.

## Experiment 3

The memory performance in Experiments 1 and 2 was analyzed based on the two-high threshold model. In both experiments, the *Pr* value – an index of how well an observer can discriminate between a new item and a repeated item – was higher for the incongruent advertisement than for congruent ones. More specifically, the participant could easily tell whether an advertisement was new or repeated if the advertisement violated the gender stereotype, and this ability was reduced if the advertisement matched the gender stereotype.

Two hypotheses are proposed to account for this effect. First, the *deep encoding hypothesis* proposes that stereotype-incongruent advertisements were encoded more deeply in the training stage, resulting in superior recognition performance compared with congruent advertisements in the testing stage. This hypothesis was based on the assertion that mental schemas lead to shallow coding of goal-irrelevant information (Sweegers and Talamini, 2014; Sweegers et al., 2015), and therefore schema-incongruent information requires more effort to encode than schema-congruent information. Second, the *conflating familiarity and recollection hypothesis* proposes that the participants were generally more familiar with stereotype-congruent advertisements, as they were consistent with their life experiences. This hypothesis is based on dual-process models of memory, where recognition performance can be determined by the participant’s assessment of a stimulus’s familiarity or recollection of details about a past event (Atkinson and Juola, 1974; Mandler, 1980; Tulving, 1985; Yonelinas, 1994). In the case of the present study, when a participant came across a stereotype-congruent advertisement in the testing stage, it might have looked familiar, even if it was a new item. Based on this, we created an online questionnaire to survey how familiar people felt with all the advertisements used in Experiment 2 on a 7-point Likert scale. A group of 91 people (56 females, *M*_age_ = 22, *SD*_age_ = 3.22) participated in the survey, and each person rated 40 advertisements that were randomly chosen from the pool of 80 advertisements. The mean familiarity value for stereotype-congruent advertisements was 2.08, and that for the stereotype-incongruent advertisements was 1.86. Although both values were small, their difference reached statistical significance (*t*[78] = 2.80, *p* = 0.006, Cohen’s *d* = 0.63). Thus, the stereotype-congruent advertisements indeed looked more familiar to the participants. Although our main purpose was to examine the participant’s recollections for different types of advertisements, their familiarity for these advertisements might have contributed to the differential memory performance for the stereotype-congruent and stereotype-incongruent advertisements.

One way to separate familiarity and recollection is to adopt the technology of electroencephalography (EEG). An EEG component known as the *parietal old/new effect* is characterized by positivity triggered by correct recognition of an old item in comparison with correct rejection of a new item occurring between 500 and 800 ms after stimulus onset (Allan et al., 1998; Friedman and Johnson, 2000; Yonelinas, 2002; MacKenzie and Donaldson, 2007; Sweegers et al., 2015). One important feature of the parietal old/new effect is that it reflects the recollection component but not the familiarity component (Curran, 2000).

According to the deep encoding hypothesis, the EEG positivity for a repeated item should be more pronounced for a stereotype-incongruent item than for a stereotype-congruent one because the former can be encoded more deeply in the training stage. According to the conflating familiarity and recollection hypothesis, the EEG positivity for a new item should be more pronounced for a stereotype-congruent item than for a stereotype-incongruent one because the former looks more familiar and participants might misattribute the quality of familiarity with recollection.

### Method

#### Participants

In the study conducted by Sweegers et al. (2015), participants were presented with a new item, an old item consistent with their expectation, or an old item inconsistent with their expectation. Based on the *F* values provided in this article, this manipulation induced an effect size of η^2^_partial_ = 0.23 for the left-side old–new partial effect. We then conducted a power analysis with G^∗^Power 3 on the basis of the effect size of η^2^_partial_ = 0.23. A sample size of 6 was required to achieve power of 0.8.^3^ Sweegers et al. (2015) mentioned that they used 72 items for each condition (repeated, new, and lured) in the testing stage, and we assumed that 36 were consistent with the participant’s schemas and 36 were not. In our study, we used only 20 items for each condition (congruent/new, congruent/old, incongruent/new, and incongruent/old; please refer to the procedure sessions of Experiments 1 and 2). Therefore, increasing the sample size was ideal. According to the recommendation provided by Simmons et al. (2011), authors must collect at least 20 observations per cell. Thus, we recruited 20 participants.

#### Procedure

**FIGURE 4:**
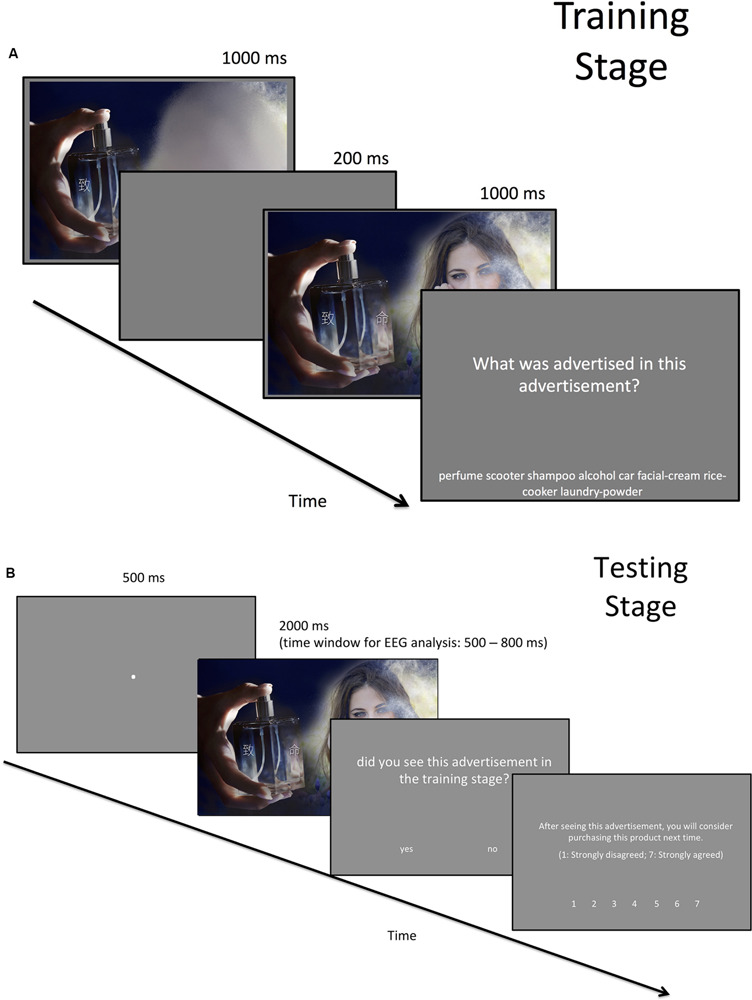
Stimuli used in the **(A)** training stage and **(B)** testing stage for Experiment 3. The presentation of the advertisement was separated into two intervals. In the first interval, the endorser’s image was blurred in order to attract the participant’s attention when the blurry part became clear in the second interval. All text was shown in Chinese in the actual experiment.

The procedure was identical to Experiment 2, except in the following ways. First, the presentation of each advertisement in the training stage was separated by two intervals lasting 1,000 ms, which were separated by a 200-ms blank. The original advertisement was shown only in the second interval, but the endorser(s) in the advertisement were blurred in the first interval so the participants could not tell whether they were female or male (Figure 4). The blur-to-clear transition could attract the participant’s attention to the endorser(s) in order to amplify the effect of gender-product type congruency. Furthermore, the total presentation time for the advertisement in the training stage was 2,000 ms, which was more than twice as long as the presentation times in Experiments 1 and 2. This helped to simplify the task. As only correctly recognized or correctly rejected trials could be included for event-related potential (ERP) analysis, we deliberately made the task easier to avoid data exclusion.

Second, this experiment was conducted in a different testing room than Experiments 1 and 2. The stimuli were shown on a VG249Q 24-in. monitor with a refresh rate of 60 Hz. The viewing distance was 60 cm. As the refresh rate was different than the previous two experiments, slightly different time parameters were used in the present experiment.

#### Image Analysis

To balance the stimuli in terms of low-level statistics, we consulted a recent study by Chiou and Lambon Ralph (2018), who used colored pictures to test the visual working memory capacity with transcranial magnetic stimulation. We followed their procedure to compute the luminance values for each pixel of each advertisement pictures used in the present study, and we averaged the pixel values to obtain the luminance index of each picture. There mean luminance values for the congruent and incongruent advertisements were not significantly different (*t*[78] = 0.48, *p* = 0.63). Furthermore, we also computed the Michelson contrast values (Michelson, 1927), defined as (*L*_max_ - *L*_min_)/(*L*_max_ + *L*_min_), where *L*_max_ and *L*_min_ represent respectively the maximum and minimum luminance values of a picture. The mean contrast values for the congruent and incongruent advertisements were not significantly different (*t*[78] = 1, *p* = 0.32).

### Data Analysis

#### Behavioral Data

As Participant Gender did not yield any significant effect or interaction in the previous experiments, it was not included as a factor for statistical analysis in this experiment. Except for the exclusion of Participant Gender, we used the identical method as that in Experiment 2 to analyze the behavioral data.

#### Electroencephalography Recording and Data Reduction

We used 32 silver/silver chloride electrodes arranged in a cap according to the International 10–20 system with the Syn-Amp2 amplifier (Compumedics Neuroscan Inc.). The sampling rate was 1,000 Hz. The impedance of each electrode was kept below 5 kΩ and was referenced to the average mastoids. All signals were processed with the Curry 8 software program (Compumedics Neuroscan Inc.). A low-pass filter of 50 Hz and a high-pass filter of 1 Hz were used, and ocular artifact correction was conducted using a regression approach (Semlitsch et al., 1986). The continuous data were segmented from −100 to 900 ms relative to experimental events of interest. For each epoch, the 100-ms period before picture onset was used for baseline correction.

To investigate the old/new effect, we consulted the study conducted by Sweegers et al. (2015), who also tested how previous knowledge or expectation affected the parietal old/new effect in a recognition task. Their EEG measurement was based on the regions of interest of electrodes P1, P3, P5, PO3, and CP3 as the left clusters and P2, P4, P6, P04, and CP4 as the right clusters. However, whereas Sweegers et al. (2015) used 64 electrodes, we had only 32 electrodes, so our left cluster was based on only P3 and CP3 and our right cluster was based on only P4 and CP4. A 300-ms window between 500 and 800 ms in these two clusters was chosen for further statistical tests.

### Results

#### Recognition

The *Pr* value did not significantly differ between the stereotype-congruent condition (*M* = 0.70) and the stereotype-incongruent condition (*M* = 0.70) (*t*[19] < 0.01, *p* > 0.99, Cohen’s *d* < 0.001).

#### Purchase Intention

Repetition had a significant effect (*F*[1, 19] = 29.61, *p* < 0.001, η^2^_partial_ = 0.61) owing to the higher purchase intention for repeated items compared with new items. For congruency, there was a trend of higher purchase intention for congruent items compared with incongruent items (*F*[1, 19] = 4.35, *p* = 0.05, η^2^_partial_ = 0.19). The interaction between repetition and congruency did not reach statistical significance (*F*[1, 19] = 0.86, *p* = 0.37, η^2^_partial_ = 0.04).

#### Electroencephalography Results

**FIGURE 5:**
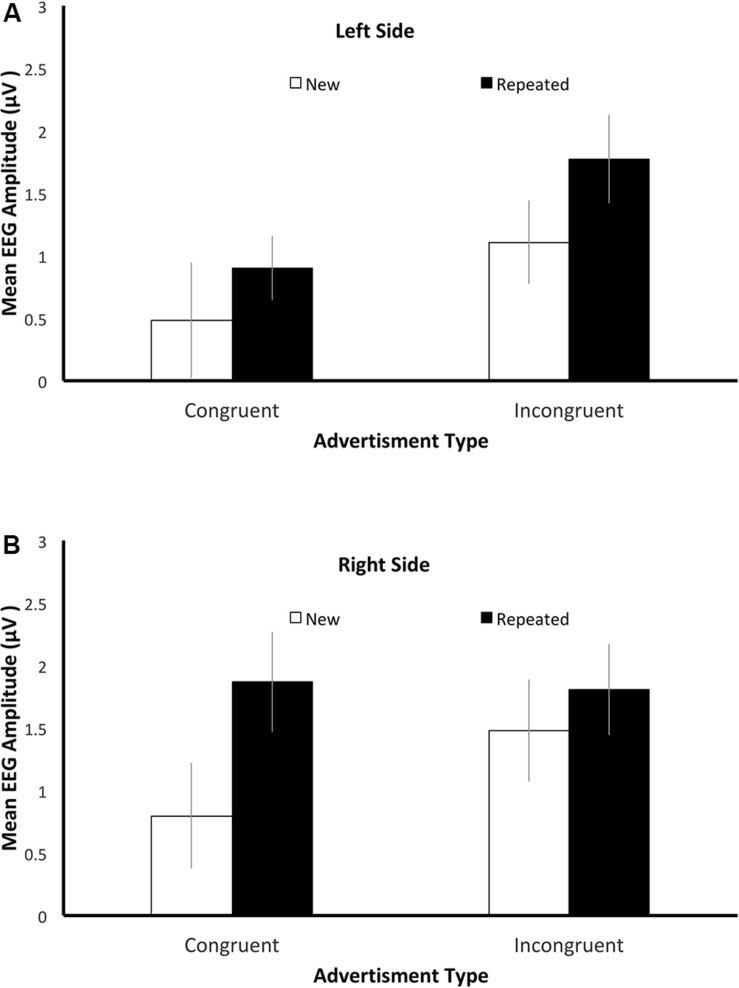
Signature of the parietal old/new effect. The mean electroencephalography (EEG) amplitudes for the **(A)** left electrodes (P3 and CP3) and **(B)** right electrodes (P4 and CP4) in the time window of 500–800 ms after stimulus onset in different conditions of Experiment 3. The error bars indicate one standard error.

We analyzed the averaged EEG amplitudes from the left clusters (P3 and CP3) and right clusters (P4 and CP4) in the time window of 500–800 ms after the onset of the advertisement in the testing stage. After the ocular artifact correction and exclusions of falsely reported trials, the average numbers of trials included in the EEG analysis were 15.8 (*SD* = 2.09), 15.35 (*SD* = 2.91), 15.95 (*SD* = 3.68), and 16.25 (*SD* = 2.31), respectively, in the repeated-congruent, repeated-incongruent, new-congruent, and new-incongruent conditions. There was no significant effect of Congruency (*F*[1, 19] = 0.02, *p* = 0.88, η^2^_partial_ = 0.001), Repetition (*F*[1, 19] = 0.65, *p* = 0.43, η^2^_partial_ = 0.03), or their interaction (*F*[1, 19] = 0.56, *p* = 0.47, η^2^_partial_ = 0.03) on the retained number of trials. The mean EEG amplitudes are shown in Figure 5. They were subjected to a repeated-measures ANOVA with factors of Congruency, Side, and Repetition. There was a significant parietal old/new effect, as evidenced by the significant effect of Repetition (*F*[1, 19] = 5.28, *p* = 0.03, η^2^_partial_ = 0.22). Higher mean amplitude was observed for the repeated advertisements (*M* = 1.59 μV) compared with new advertisements (*M* = 0.97 μV).

There was a three-way interaction among the three factors (*F*[1, 19] = 9.14, *p* = 0.007, η^2^_partial_ = 0.32). We thus conducted simple main effect analyses. To examine the deep encoding hypothesis and the conflating familiarity and recollection hypothesis, we separated the dataset into the “repeated” subset, which was composed of only the trials with repeated advertisements, and the “new” subset, which was composed of only the trials with new advertisements. For the repeated subset, there was a significant interaction between Side and Congruency (*F*[1, 19] = 13.19, *p* = 0.002, η^2^_partial_ = 0.41). We then examined the effect of Congruency separately for the left and right sides. We found that it had a significant effect for the left side (*t*[19] = 3.04, *p* = 0.007, Cohen’s *d* = 0.68) caused by the higher amplitude for repeated-incongruent advertisement (*M* = 1.77 μV) than for repeated-congruent ones (*M* = 0.90 μV). For the right side, the effect of Congruency was not significant (*t*[19] = 0.16, *p* = *0.87*, Cohen’s *d* = 0.04). The waveform of the left parietal electrodes supported the deep encoding hypothesis; because incongruent advertisements were encoded more deeply, they provoked higher positivity than congruent ones.

With regard to the new subset, there was no significant effect of Congruency (*F*[1, 19] = 2.87, *p* = 0.11, η^2^_partial_ = 0.13), Side (*F*[1, 19] = 1.78, *p* = 0.20, η^2^_partial_ = 0.09), or interaction between the two (*F*[1, 19] = 0.07, *p* = 0.79, η^2^_partial_ = 0.004). Therefore, the conflating familiarity and recollection hypothesis was not supported, as the new-congruent advertisements did not provoke higher positivity than new-incongruent ones. According to the rating results collected from a separate group of participants, the participants might have felt that the new-congruent advertisements looked more familiar than new-incongruent ones. Despite this, their ERP recordings suggested that familiarity did not contribute to their recollection. In fact, the amplitude for the congruent advertisements tended to be lower than that for the incongruent ones (Figure 5).

**FIGURE 6:**
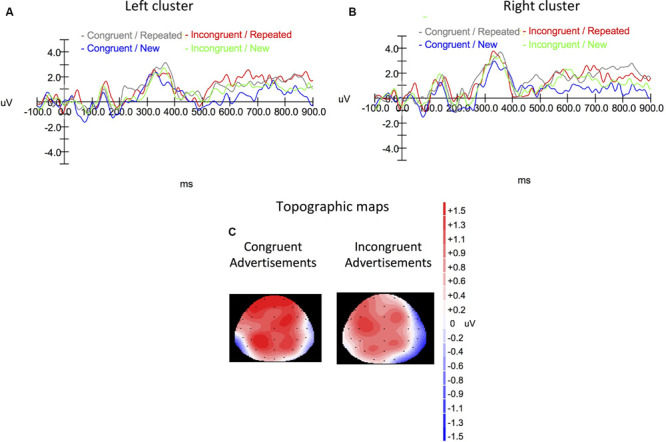
The average event-related potential (ERP) waveforms from the **(A)** left cluster (P3, CP3) and the **(B)** right cluster (P4, CP4) for the four experimental conditions. **(C)** Topographic maps of amplitude differences between the congruent-repeated vs congruent-new condition (left), and incongruent repeated vs incongruent-new condition (right).

Complete illustrations for the ERP waveforms in the time window from -100 to 900 ms for the left and right hemispheres are shown in Figures 6A, B, respectively. Topographic maps for the time window of 500–800 ms are shown in Figure 6C.

### Discussion

The behavioral data in this experiment were not consistent with those of previous experiments, as stereotype congruency did not have a significant effect on *Pr*. However, it is worth noting that the stimulus presentation time in the training stage was 2,000 ms in the current experiment, which was twice as long as the presentation time in previous experiments. Our original goal was to simplify the task to include as many correctly recognized and correctly rejected trials as possible for ERP analysis, but this strategy led to a ceiling effect in the behavioral data. Nevertheless, the ERP signals still revealed a significant interaction among Congruency, Side, and Repetition. A significant effect of congruency was measured from the left electrodes for the repeated advertisements, which supported the deep encoding hypothesis as an incongruent advertisement was encoded more deeply than a congruent one. The lateralization of the parietal positivity to the left side was consistent with previous studies (Schloerscheidt and Rugg, 1997; Wilding, 2000; Finnigan et al., 2002).

## General Discussion

### Summary of Results

The first two experiments were conducted to test the effect of gender stereotype congruency on memory and purchase intention. Real advertisements (Experiment 1) and fabricated advertisements (Experiment 2) were used. Some were stereotype congruent (e.g., a male endorser for a car), and some were stereotype incongruent (e.g., a female endorser for a car). Both experiments showed a memory benefit for stereotype-incongruent items. In addition, stereotype-congruent advertisements provoked higher purchase intention than stereotype-incongruent ones in Experiment 1, which used real advertisements, but not in Experiment 2, which used fabricated advertisements. In Experiment 3, which measured EEG signals, significantly larger positive amplitudes were found for incongruent repeated advertisements than congruent repeated advertisements. This supported the deep encoding hypothesis, which claims that incongruent advertisements are encoded more deeply than congruent ones during the training stage. The following sections discuss gender stereotype and its effect on memory, purchase intention, and ERP signals.

### Effect of Stereotype Congruency on Memory

The results of the present study, which indicate a memory benefit for stereotype-incongruent advertisements due to their higher discriminability, were consistent with those of previous studies that showed a memory benefit for surprising or atypical events or faces (Graesser et al., 1980; Shapiro and Penrod, 1986; Schmidt, 1991; Stangor and McMillan, 1992; Erdfelder and Bredenkamp, 1998). Possibly, compared with typical stimuli, atypical stimuli require more effort to encode (Sweegers and Talamini, 2014; Sweegers et al., 2015) and thus lead to better memory performance. The EEG results of Experiment 3 support this assertion. However, the results of the present study were inconsistent with a series of studies that showed a memory benefit for events that were consistent with participants’ schemas (Castel, 2005; Kleider et al., 2012; Malodia et al., 2017). It is possible that these results are due to the use of the cued recall task for memory measurement in these studies, which may have allowed participants to use their stereotypes or schemas to infer instead of recall in their responses. A recognition task was used in the present study to avoid stereotype-induced inference.

From a neuro-scientific approach, van Kesteren et al. (2012) proposed the schema-linked interactions between medial prefrontal and medial temporal regions (SLIMM) model. In their model, the medial temporal lobe (MTL) and medial prefrontal cortex (mPFC) are highly involved in the formation and retrieval of mental representations. The MTL includes many subregions that are conventionally regarded as highly related to the formation of episodic memories, such as *hippocampus*, *perirhinal* and *entorhinal cortices*, and *parahippocampal gyrus*. Research has shown that the novelty of information is highly correlated with the activities of the MTL (Kirchhoff et al., 2000; Kumaran and Maguire, 2007; Fenker et al., 2008). In consequence, novelty improves the formation of episodic memories. According to the results of the present study, novelty could be triggered by presenting information that violates one’s gender stereotype.

As our measurement of memory relied on participants’ subjective reports, attention played an important role in selecting and filtering the information used for conscious reports. We used two different stimulus presentation times in Experiments 1 and 2 (83 and 977 ms) in order to apply the two-stage model of attentional processing (Kanwisher, 1987; Chun and Potter, 1995; Bowman and Wyble, 2007; Goodbourn and Holcombe, 2015; Lo and Yeh, 2018; Lo and Wang, 2019). In general, memory performance improved with the long presentation time because more information could pass through the attentional selection mechanism and reach participants’ consciousness. The lack of significant interaction between stimulus duration and memory benefit for incongruent advertisements suggested that stimulus novelty played a similar facilitative role in the two stages.

### Effect of Stereotype Congruency on Purchase Intention

Cognitive psychologists tend to adopt an information-processing view to analyze human behavior, and therefore memory performance is often used an index for behavior efficiency. In the advertising literature, however, researchers are more concerned with attitude or purchase intention (Kamins, 1990; Kamins and Kamal, 1994; Ryu et al., 2006; Törn, 2012; Harmon-Kizer, 2017). Attitude is a multifaceted construct that is composed of affect, behavior, and cognition (Eagly and Chaiken, 1998; Van den Berg et al., 2006). Thus, to avoid potential complexity, we chose to measure purchase intention directly instead of measuring attitude. In Experiment 1, the participants’ purchase intention was higher for stereotype-congruent advertisements than incongruent ones, consistent with studies showing a schema-congruency benefit (Kamins, 1990; Till and Busler, 2000; Lee and Thorson, 2008). However, the effect became insignificant with the fabricated advertisements in Experiment 2 and only marginally significant in Experiment 3. We suspected that participants’ previous exposure to gender stereotype messages in the media might be the cause of this result. Gender stereotypes can be encountered in different advertisements; for example, voluptuous women may endorse product types related to body care, or macho men may endorse product types related to power and speed. This strategy is prevalent in Taiwanese society (Tsai, 2010). As the media play an important role in changing attitudes (Mahler et al., 2010), our participants might have learned that a stereotype-congruent association between a celebrity endorser and a real product predicted good usage experiences, so their purchase intention in Experiment 1 may have reflected their prior experiences. In Experiments 2 and 3, all the endorsers and products were new to the participants, which may not have provoked as many memories as the advertisements with celebrity endorsers, therefore reducing the stereotype effect.

In all of the three experiments, the participants indicated higher purchase intention for products of repeated advertisements than new advertisements. This could be due to the psychological phenomenon of *mere exposure effect* (Zajonc, 2001): People tend to develop a preference for things they have been exposed to. In Experiment 1, the mere exposure effect was more pronounced with a long presentation time than a short one. With a short presentation time, the participants’ purchase intention demonstrated a mere exposure effect; as the presentation time increased, the participants could clearly recognized the celebrity endorsers in the advertisements, and the mere exposure effect was intensified by the participants’ familiarity for the endorsers.

### Effect of Stereotype Congruency on Event-Related Potential Signals

The participants’ performance on the recognition task might reflect a mixture of the familiarity effect and the recollection effect. Thus, we used the parietal old/new effect to specifically examine the effect of gender stereotype on recollection. In general, the EEG signals were more positive for repeated advertisements than for new advertisements, as evidenced by the main effect of Repetition in Experiment 3. This observation replicated the parietal old/new effect (Allan et al., 1998; Friedman and Johnson, 2000; Yonelinas, 2002; MacKenzie and Donaldson, 2007; Sweegers et al., 2015). Further comparison of the waveforms induced by repeated items and new items suggested that repeated incongruent advertisements induced even higher amplitudes than repeated congruent advertisements, but only on the left side.

In previous studies, there was a tendency for a left lateralized parietal old/new effect to appear in memory tasks involving semantic processing (Schloerscheidt and Rugg, 1997; Wilding, 2000; Finnigan et al., 2002) but a right lateralized parietal old/new effect to be observed in tasks involving spatial processing. For example, in the study of Sweegers et al. (2015), the participants had to focus on the relationship between houses and faces, and a more pronounced positive effect for incongruent information (as compared with congruent information) was observed on the right side. It is possible that the processing of spatial information related to houses and faces involved activities in the right hemisphere. In the present study, however, we manipulated the consistency between the endorser’s gender and the product type, which involved mainly semantic processing, and for semantic or linguistic processing, the left hemisphere should be more dominant.

### Limitations and Future Directions

In this study, we aimed to integrate the disciplines of cognitive psychology, neuroscience, and advertising research to examine how gender stereotypes modulate memory and behavior. However, from a psychological perspective, the stimuli used in this study might not have been well controlled. Experiment 1 could be viewed as an exploratory study, so the advertisements were not very strictly controlled. In Experiments 2 and 3, we tried our best to control their low-level features, such as luminance and contrast, and all the advertisements were created in our lab so participants should not have been exposed to them previously. Still, there might be some other potential confounding factors that future researchers could design more sophisticate experiments to rule out.

From a neuro-scientific perspective, the EEG waveforms observed in Experiment 3 appeared to be noisy, as one can see from Figure 6. Our main purpose for the EEG experiment was to examine the deep encoding hypothesis, which relied on the comparison between the repeated-incongruent advertisements and repeated-congruent ones. For this comparison, the effect size index Cohen’s *d* was 0.68, which was considered to be between medium and large (Cohen, 1988). Despite the substantial effect size, the waveforms still looked noisy. Future research with larger numbers of observations per condition is required to ensure the reliability of the deep-encoding account for the parietal old/new effect.

From the perspective of advertising research, the materials used in the present study might be too limited. We only used eight different product types, and the research outcomes might not be generalizable to other types of products or services. Furthermore, the measurement of purchase intention was not authentic because the participants did not truly have to purchase anything. It is impossible to offer real products for participants to purchase in a laboratory study, but it would be possible to create a scenario in which the participants have a certain amount of simulated funds and must make a purchase decision (e.g., a gift). When given a more realistic scenario, the participants’ behavior in a laboratory setting might be more similar to their true behaviors.

In both behavioral experiments (Experiments 1 and 2), Participant Gender did not moderate the effect of Congruency, as evidenced by the lack of significant interaction between Participant Gender and Congruency. Possibly, the social cultural influences of gender stereotypes on females and males are equivalent, so they demonstrated similar susceptibility to gender stereotypes. However, a lack of statistical significance does not necessarily guarantee a lack of effect. For future researchers who focus on the gender effect on gender stereotypes, a larger sample is required to increase the statistical power. Furthermore, a quasi-experimental design is a preferred method to study the effect of Participant Gender. In a quasi-experimental design, participants in the female group and male group should be comparable in age, socioeconomic status, political ideology, and so forth. Therefore, any observed difference between the female group and the male group can be inferred to be due to the participant’s gender, instead of other factors that potentially covary with gender.

### Conclusion

In sum, we adopted a psychological perspective to examine the effect of gender stereotypes on memory and purchase intention, and we found a memory benefit for information that is incongruent with gender stereotypes. This memory benefit is due to the deep encoding of incongruent information, as shown by the EEG results in Experiment 3. Regarding purchase intention, a benefit for congruent advertisements was observed only with celebrity endorsers, and not for unknown endorsers. As persuasion involves conveying important messages and branding those messages into audiences’ minds, memory performance should be the main index for persuasion successfulness. Regarding gender-related issues, the present study offers evidence that images violating gender stereotypes could actually enhance audiences’ memory.

## Data Availability Statement

The datasets generated for this study are available on request to the corresponding author.

## Ethics Statement

The studies involving human participants were reviewed and approved by Research Ethics Committee for Human Subject Protection, National Chiao Tung University. The patients/participants provided their written informed consent to participate in this study.

## Author Contributions

S-YL conceived this study. S-YL and J-TK designed the study and analyzed the data. All authors contributed to the drafting and editing of the manuscript and approved the submitted version.

## Conflict of Interest

The authors declare that the research was conducted in the absence of any commercial or financial relationships that could be construed as a potential conflict of interest.

## Footnotes

1 For this analysis, the statistical test was set to “ANOVA: Repeated measures, within factors.” The number of groups was set to 1, the number of measurements was set to 2 (congruent/incongruent), the correlation between repeated measurements was set to 0.5, and the non-sphericity correction was set to 1.

2 https://www.shutterstock.com/

3 For this analysis, the statistical test was set to “ANOVA: Repeated measures, within factors.” The number of groups was set to 1, the number of measurements was set to 4 (old-congruent/old-incongruent/new-congruent/new-incongruent), the correlation between repeated measurements was set to 0.5, and the non-sphericity correction was set to 1.

## Supplementary Material

DATA SHEET S1 Appendix – images used in the study.
