# Supplementary material for: How Does Gender Stereotype Affect the Memory of Advertisements? A Behavioral and Electroencephalography Study
Source: Front Psychol. 2020 Jul 14;11:1580. doi: 10.3389/fpsyg.2020.01580 (PMC7381247; doi:10.3389/fpsyg.2020.01580)
Supplement: DATA SHEET S1 — Appendix – images used in the study. [file Data_Sheet_1.pdf]

Appendix

Stereotype-congruent advertisements

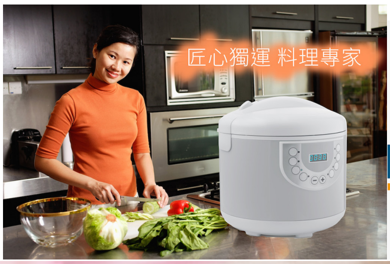

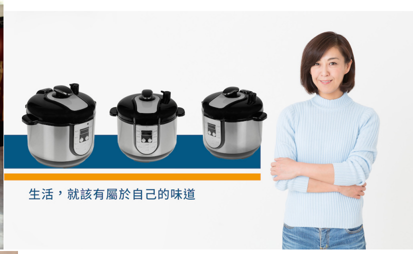

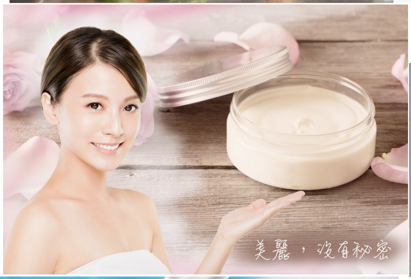

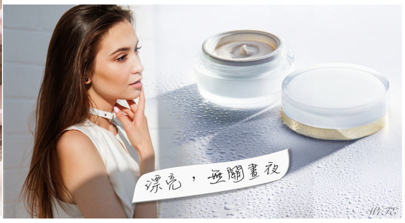

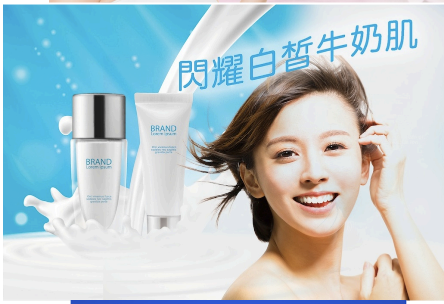

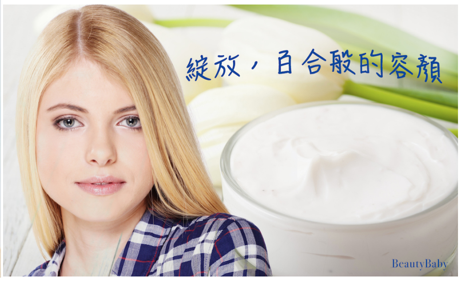

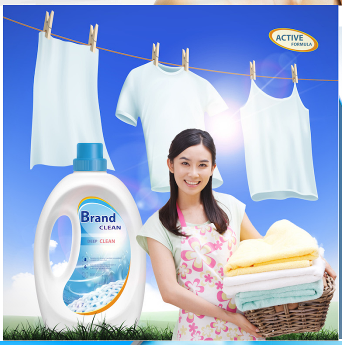

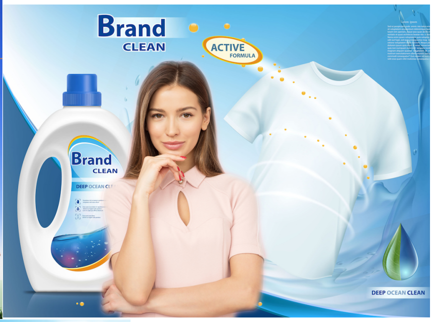

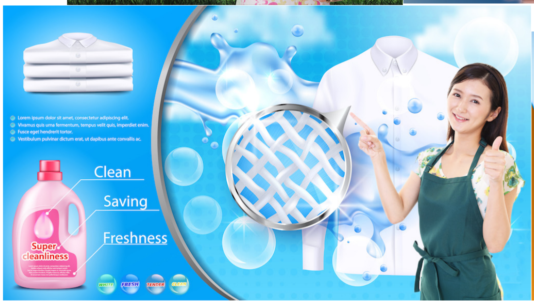

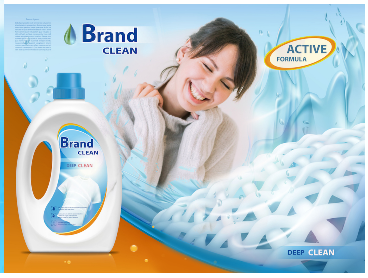

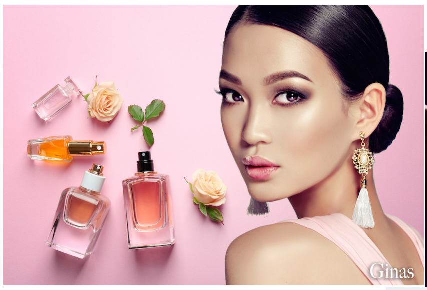
Ginas
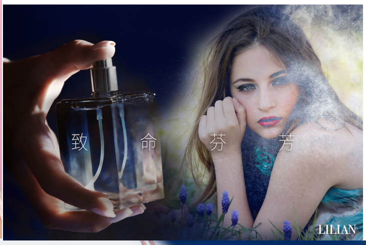
致 命 芬 芳
LILIAN
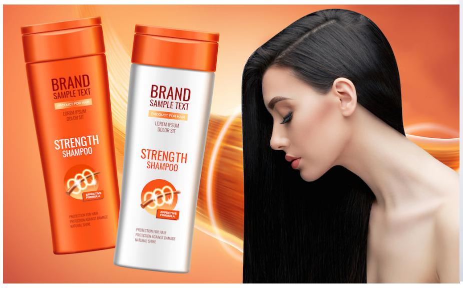
BRAND
SAMPLE TEXT
STRENGTH
SHAMPOO
BRAND
SAMPLE TEXT
STRENGTH
SHAMPOO
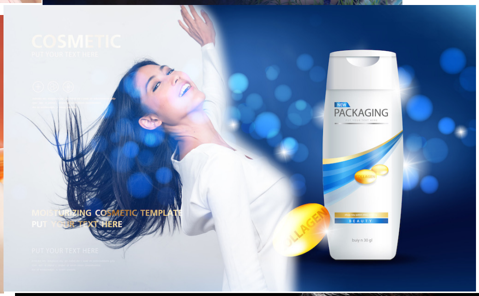
COSMETIC
PACKAGING
MOISTURIZING COSMETIC TEMPLATE
PUT YOUR TEXT HERE
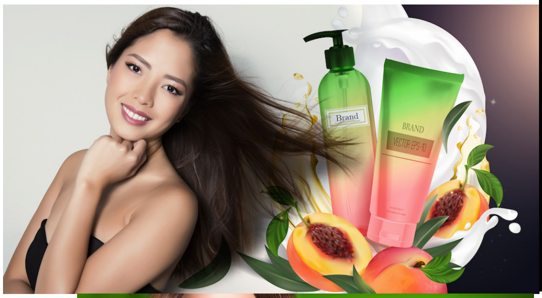
Brand
BRAND
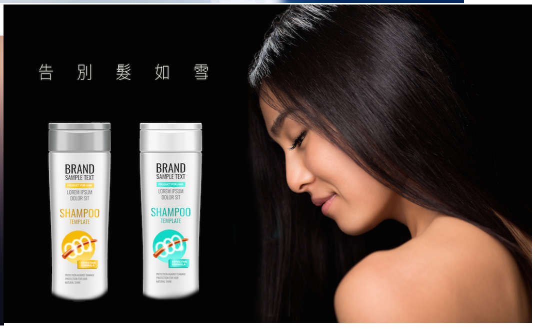
告 别 髮 如 雪
BRAND
SAMPLE TEXT
SHAMPOO
TEMPLATE
BRAND
SAMPLE TEXT
SHAMPOO
TEMPLATE
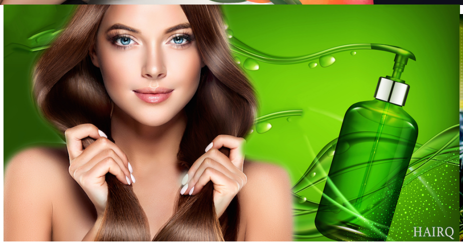
HAIRQ
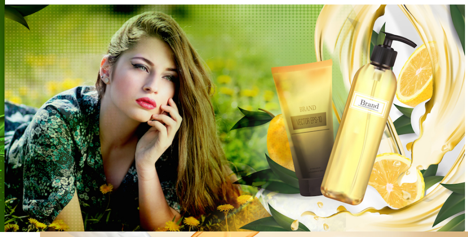
Brand
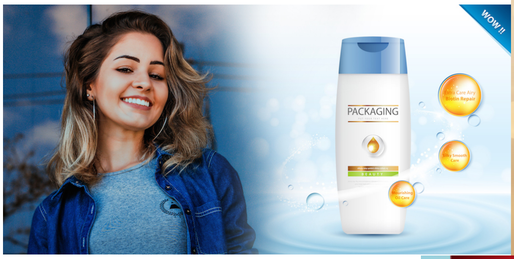
WOW !!
PACKAGING
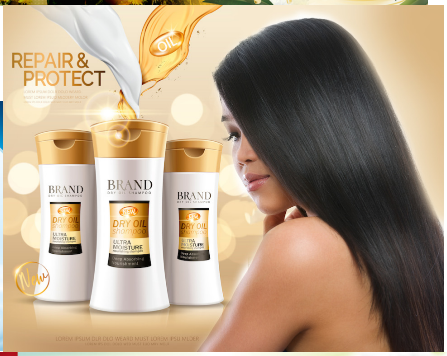
REPAIR &
PROTECT
OIL
BRAND
DRY OIL
BRAND
DRY OIL
BRAND
DRY OIL
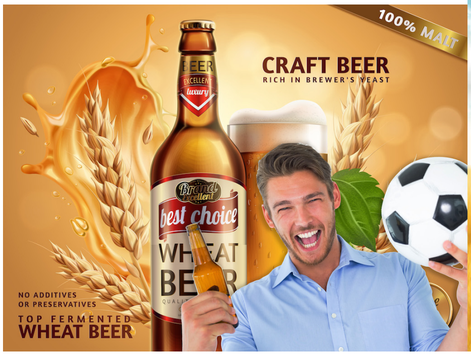
100% MALT
CRAFT BEER
RICH IN BREWER'S YEAST
best choice
WHEAT
BEER
NO ADDITIVES
OR PRESERVATIVES
TOP FERMENTED
WHEAT BEER
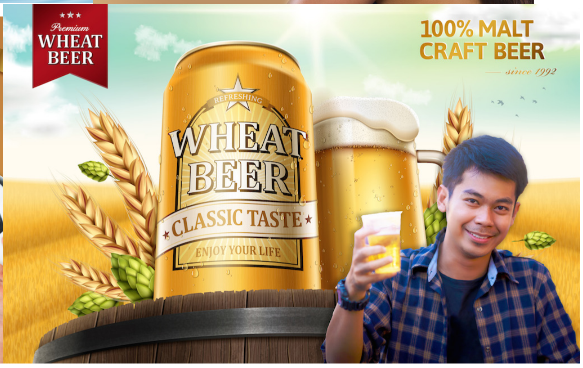
Premium
WHEAT
BEER
100% MALT
CRAFT BEER
since 1992
REFRESHING
WHEAT
BEER
CLASSIC TASTE
ENJOY YOUR LIFE

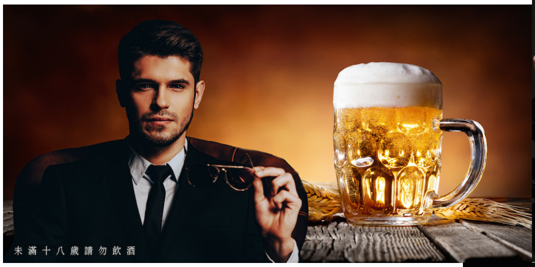
未滿十八歲請勿飲酒

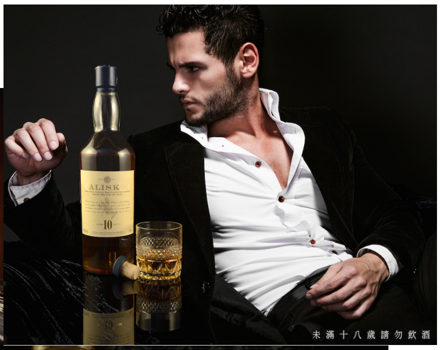
未滿十八歲請勿飲酒

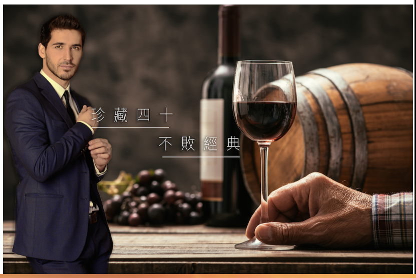
珍藏四十
不敗經典

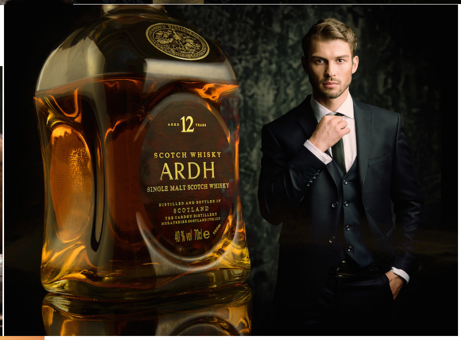
12
SCOTCH WHISKY
ARDH
SINGLE MALT SCOTCH WHISKY
SCOTLAND

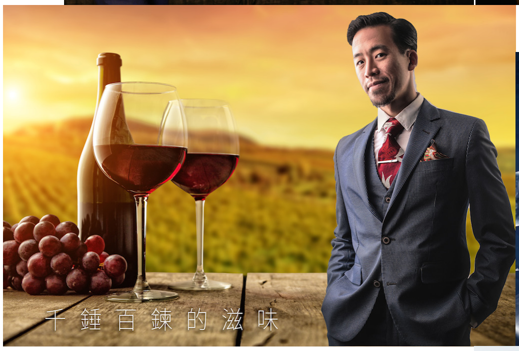
千錘百鍊的滋味

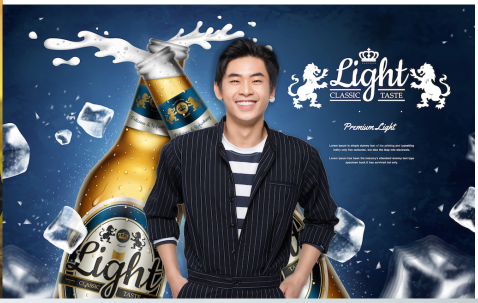
Light
CLASSIC TASTE
Premium Light

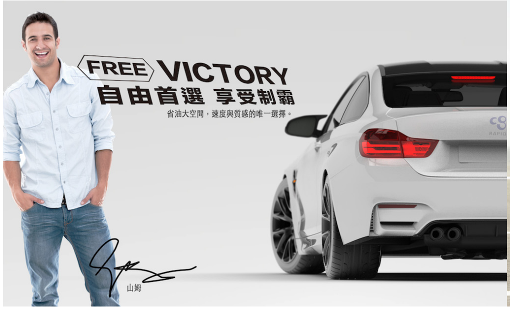
FREE VICTORY
自由首選 享受制霸
省油大空間，速度與質感的唯一選擇。
山姆

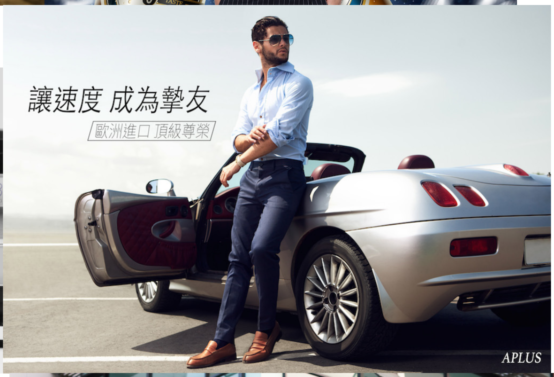
讓速度 成為摯友
歐洲進口 頂級尊榮
APLUS

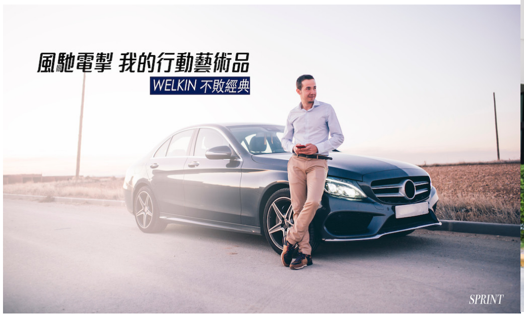
風馳電掣 我的行動藝術品
WELKIN 不敗經典
SPRINT

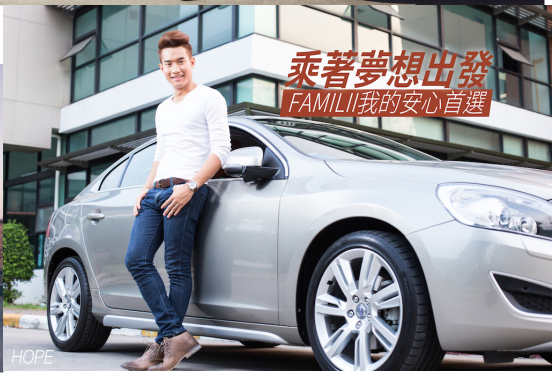
乘著夢想出發
FAMILI我的安心首選
HOPE

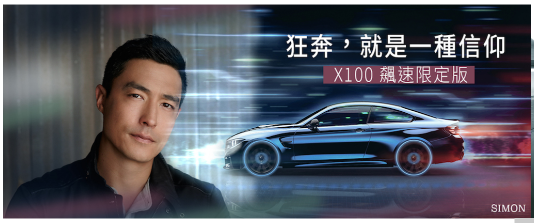
狂奔，就是一種信仰
X100 飆速限定版
SIMON

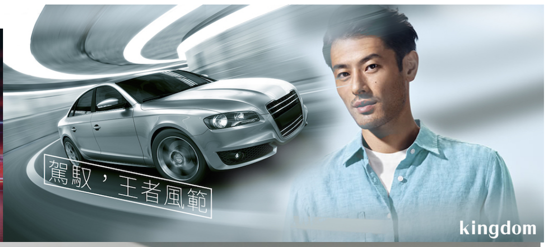
駕馭，王者風範
kingdom

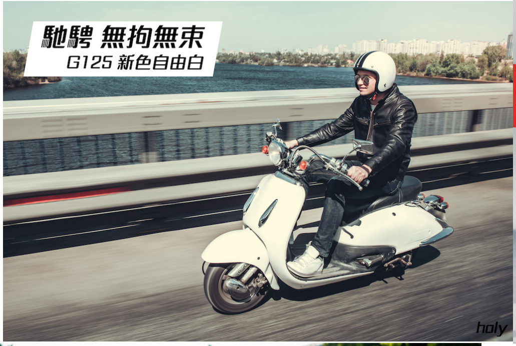
馳騁 無拘無束
G125 新色自由白
holy

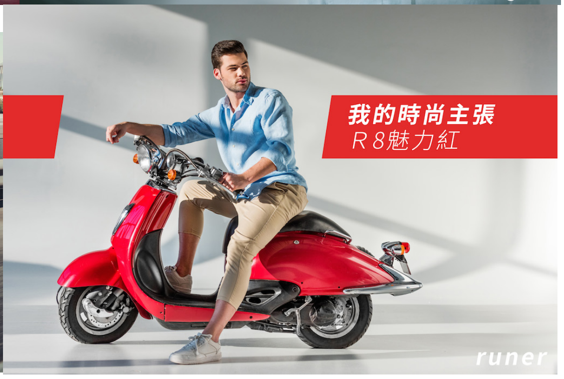
我的時尚主張
R 8魅力紅
runer

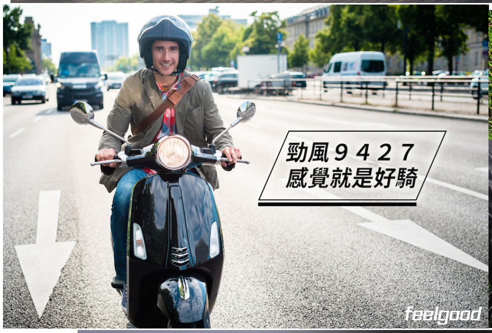
勁風 9 4 2 7
感覺就是好騎
feelgood

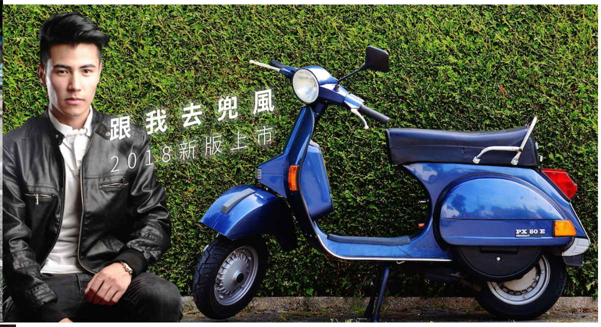
跟 我 去 兜 風
2 0 1 8 新 版 上 市

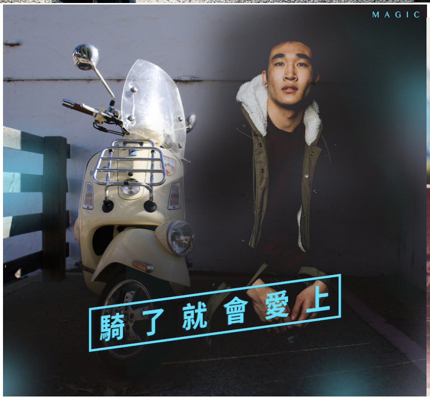
MAGIC
騎 了 就 會 愛 上

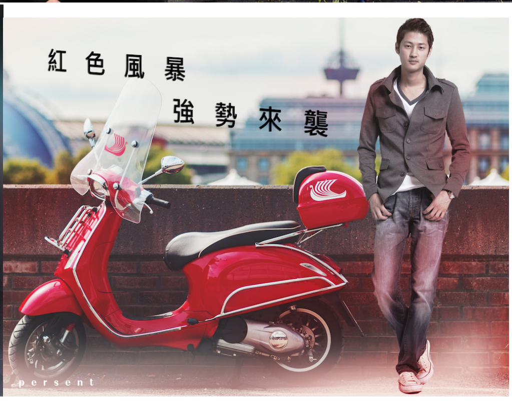
紅 色 風 暴
強 勢 來 襲
persent

Stereotype-incongruent advertisements

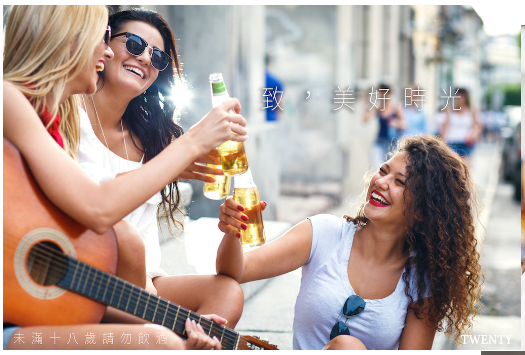

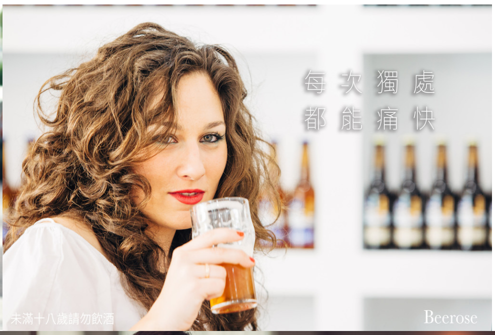

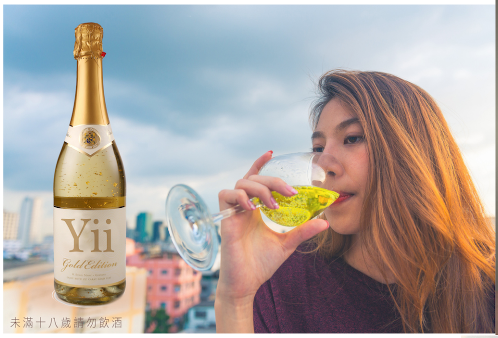

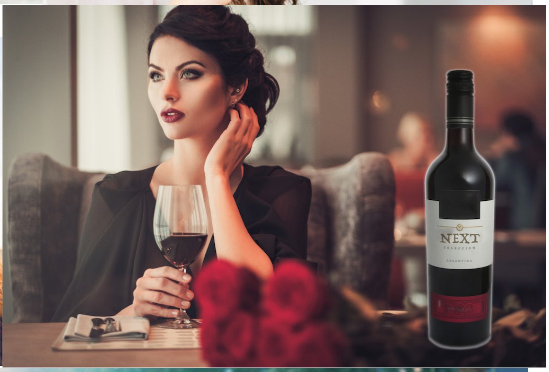

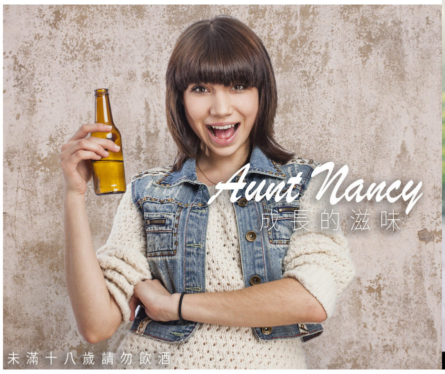

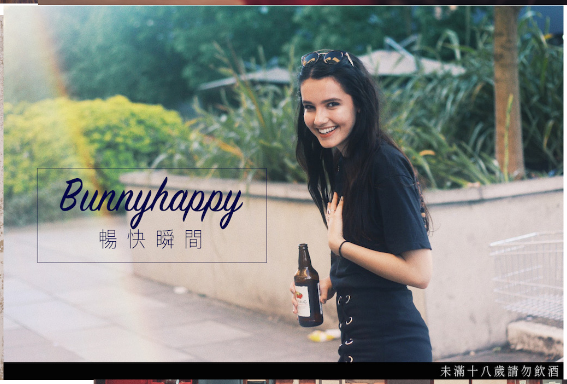

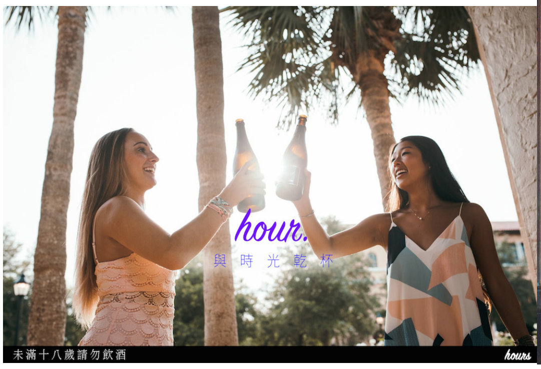

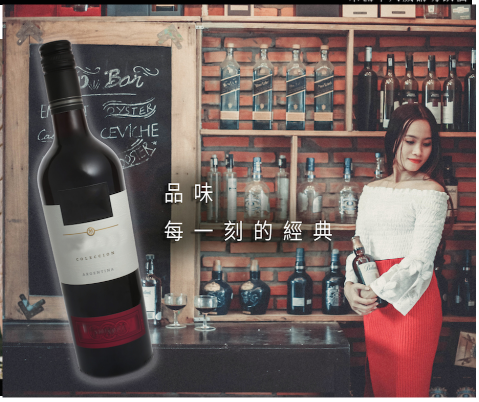

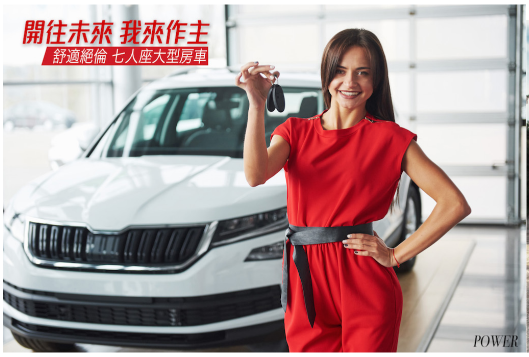
開往未來 我來作主
舒適絕倫 七人座大型房車
POWER
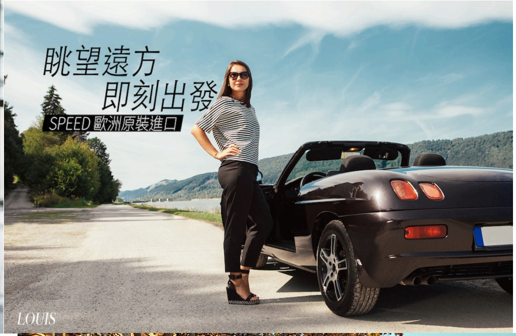
眺望遠方
即刻出發
SPEED 歐洲原裝進口
LOUIS
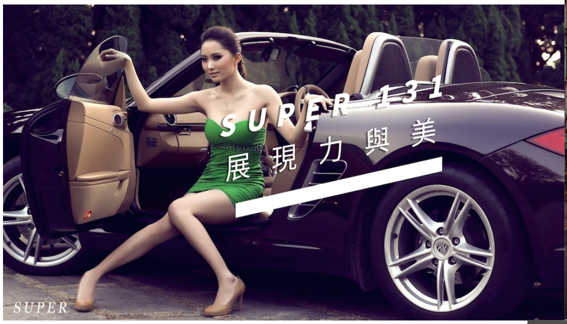
SUPER 131
展 現 力 與 美
SUPER
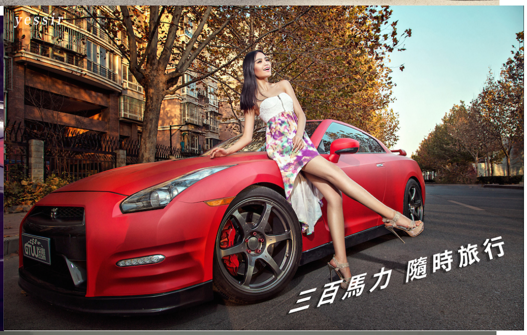
三百馬力 隨時旅行
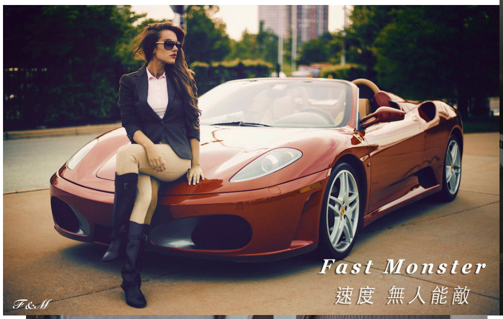
Fast Monster
速度 無人能敵
F&M
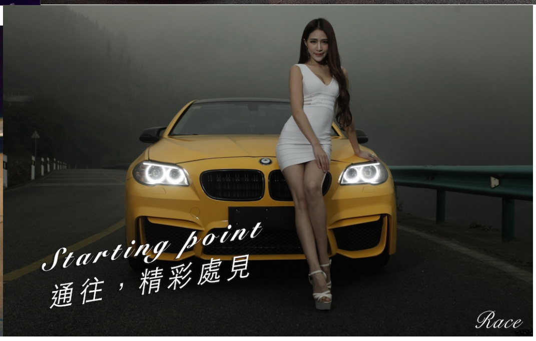
Starting point
通往，精彩處見
Race
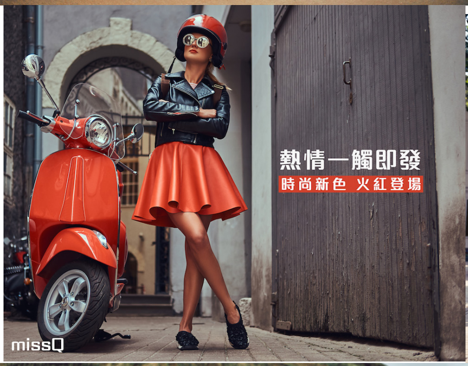
熱情一觸即發
時尚新色 火紅登場
missQ
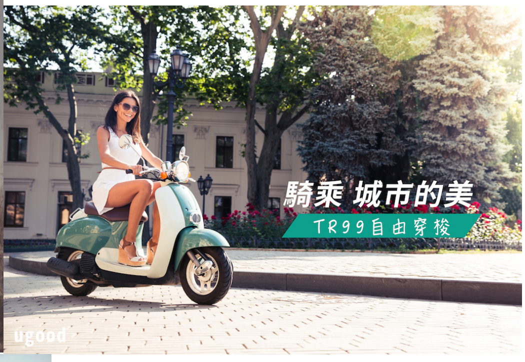
騎乘 城市的美
TR99自由穿梭
ugood
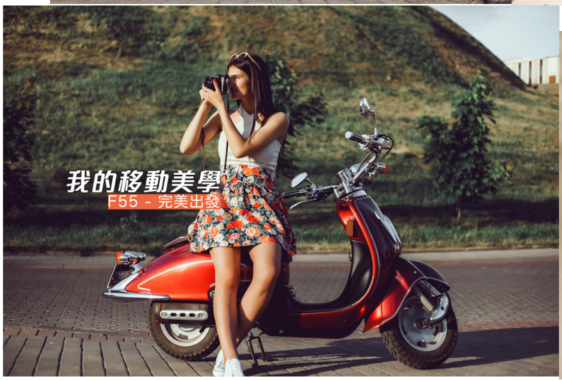
我的移動美學
F55 - 完美出發
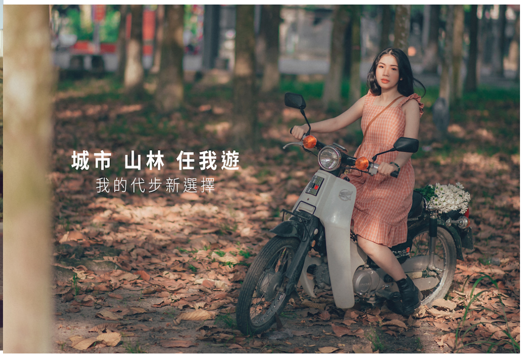
城市 山林 任我遊
我的代步新選擇

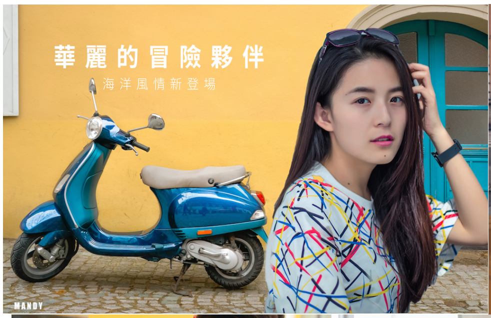
華麗的冒險夥伴
海洋風情新登場
MANDY

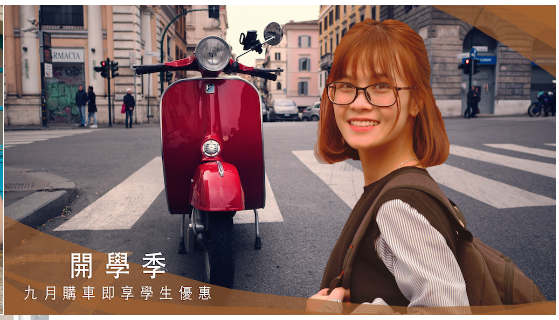
開學季
九月購車即享學生優惠

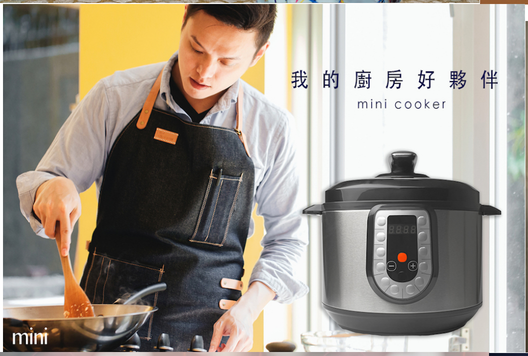
我的廚房好夥伴
mini cooker
mini

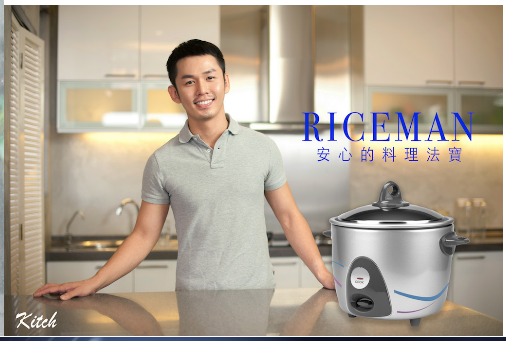
RICEMAN
安心的料理法寶
Kitch

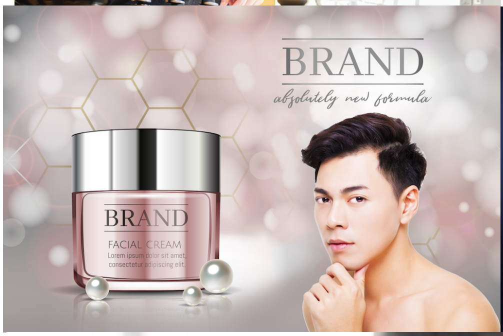
BRAND
absolutely new formula
BRAND
FACIAL CREAM

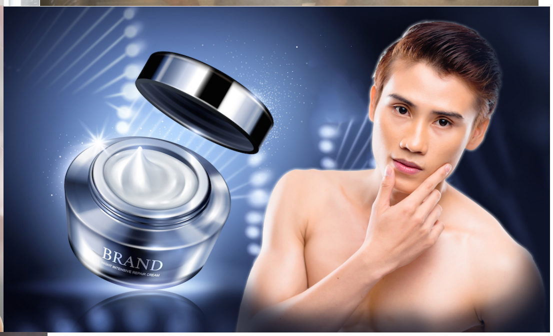
BRAND

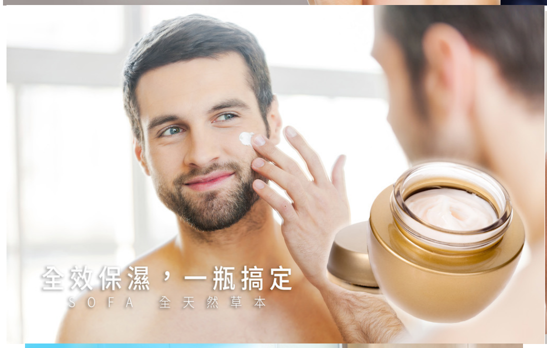
全效保濕，一瓶搞定
SOFA 全天然草本

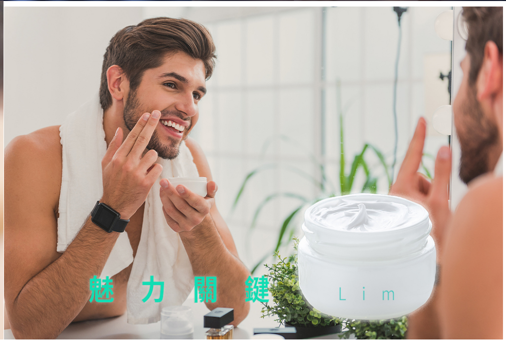
魅力關鍵
Lim

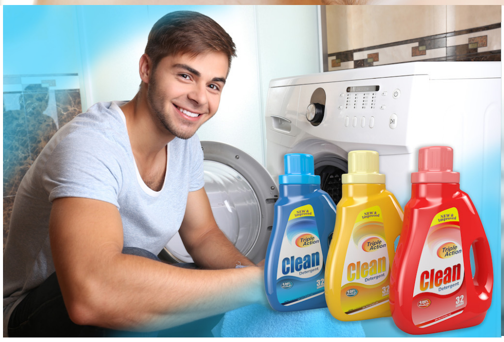
Clean
Clean
Clean

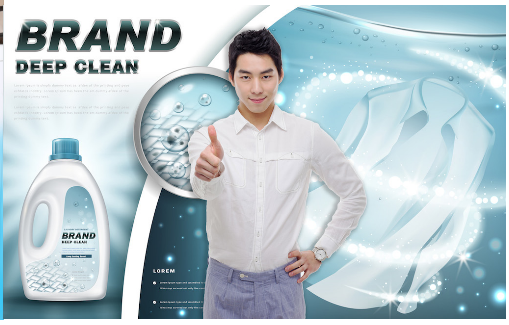
BRAND
DEEP CLEAN
BRAND

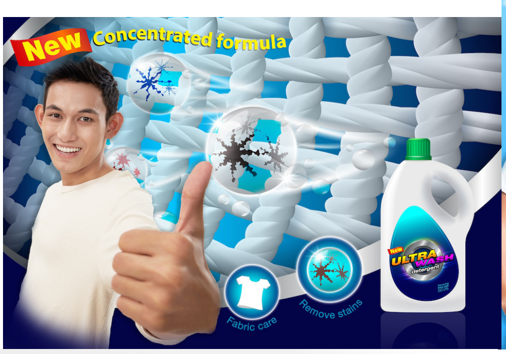
New
Concentrated formula
Fabric care
Remove stains

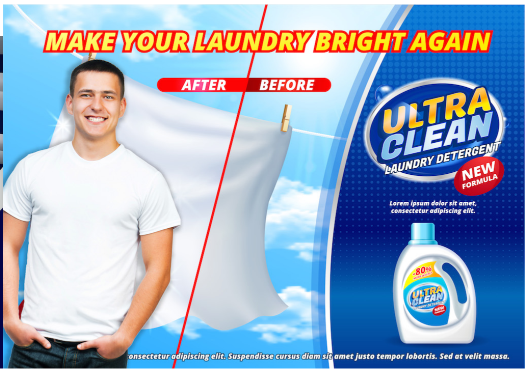
MAKE YOUR LAUNDRY BRIGHT AGAIN
AFTER
BEFORE
ULTRA CLEAN
LAUNDRY DETERGENT
NEW FORMULA
Lorem ipsum dolor sit amet, consectetur adipiscing elit.

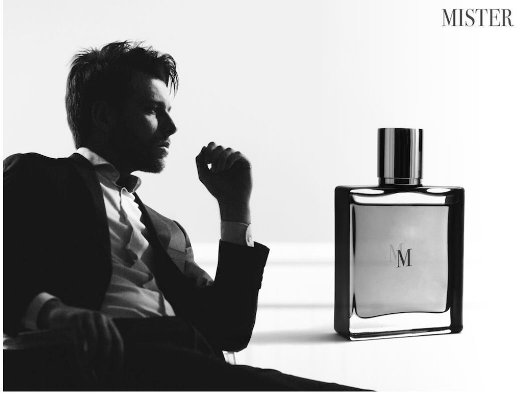
MISTER

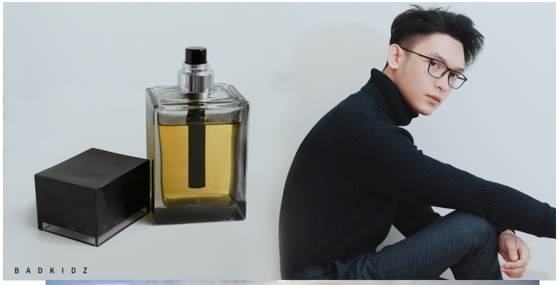
B A D K I D Z

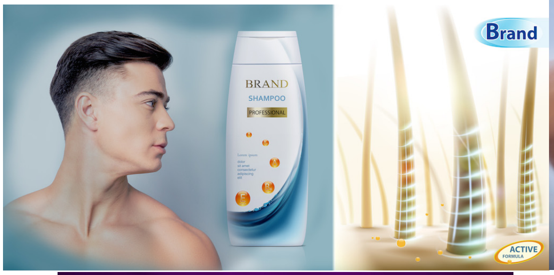
BRAND
SHAMPOO
PROFESSIONAL
Brand
ACTIVE
FORMULA

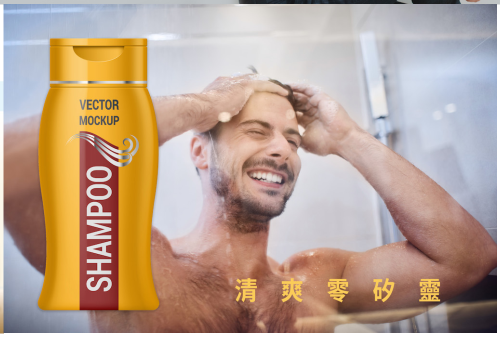
VECTOR
MOCKUP
SHAMPOO
清 爽 零 矽 靈

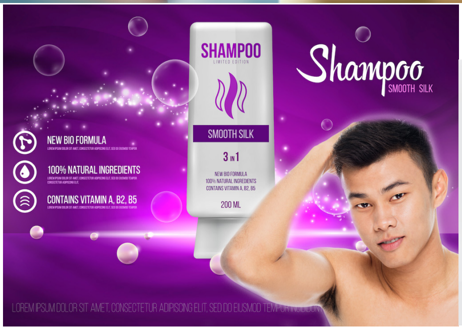
SHAMPOO
SMOOTH SILK
3 IN 1
200 ML
Shampoo
SMOOTH SILK
NEW BIO FORMULA
100% NATURAL INGREDIENTS
CONTAINS VITAMIN A, B2, B5

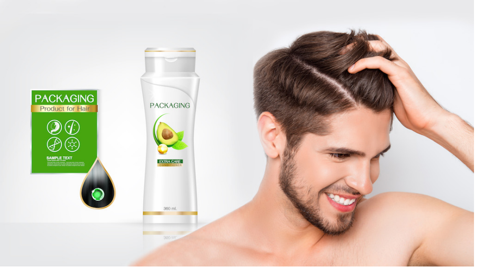
PACKAGING
Product for Hair
PACKAGING

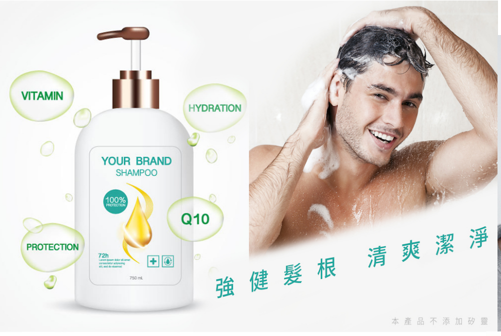
VITAMIN
HYDRATION
YOUR BRAND
SHAMPOO
Q10
PROTECTION
強 健 髮 根　清 爽 潔 淨

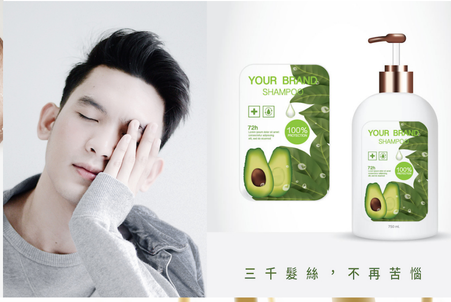
YOUR BRAND
SHAMPOO
三 千 髮 絲 ， 不 再 苦 惱

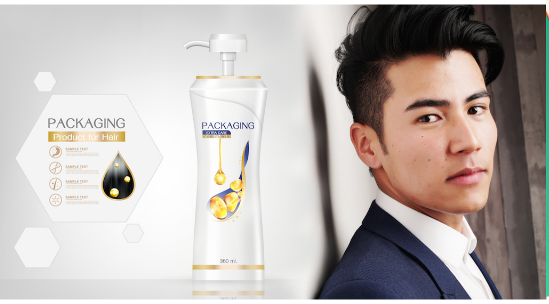
PACKAGING
Product for Hair
PACKAGING

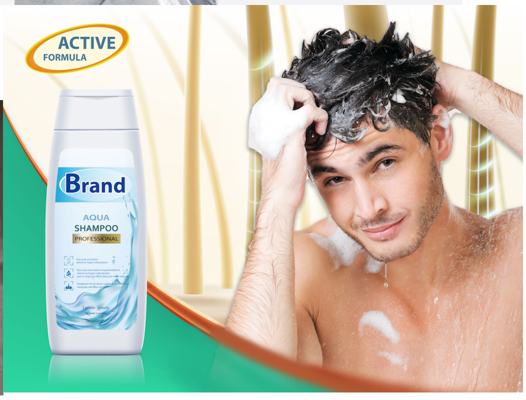
ACTIVE
FORMULA
Brand
AQUA
SHAMPOO
PROFESSIONAL
